# Self-templated synthesis of uniform hollow spheres based on highly conjugated three-dimensional covalent organic frameworks

**DOI:** 10.1038/s41467-020-18844-4

**Published:** 2020-11-03

**Authors:** Yuan-Yuan Liu, Xiang-Chun Li, Shi Wang, Tao Cheng, Huiyan Yang, Chen Liu, Yanting Gong, Wen-Yong Lai, Wei Huang

**Affiliations:** 1 Key Laboratory for Organic Electronics and Information Displays & Institute of Advanced Materials (IAM), Nanjing University of Posts & Telecommunications, 9 Wenyuan Road, 210023 Nanjing, China; 2 Frontiers Science Center for Flexible Electronics (FSCFE), MIIT Key Laboratory of Flexible Electronics (KLoFE), Northwestern Polytechnical University, West Youyi Road, 710072 Xi’an, China

**Keywords:** Metal-organic frameworks, Porous materials

## Abstract

Covalent organic frameworks (COFs) have served as a family of porous crystalline molecules for various promising applications. However, controllable synthesis of COFs with uniform morphology is paramount yet still remains quite challenging. Herein, we report self-templated synthesis of uniform and unique hollow spheres based on highly conjugated three-dimensional (3D) COFs with diameters of 500–700 nm. A detailed time-dependent study reveals the continuous transformation from initial nano sphere-like particles into uniform hollow spherical structures with Ostwald ripening mechanism. Particularly, the resulting 3D COF (3D-Sp-COF) is prone to transport ions more efficiently and the lithium-ion transference number (*t*^+^) of 3D-Sp-COF reaches 0.7, which even overwhelms most typical PEO-based polymer electrolytes. Inspiringly, the hollow spherical structures show enhanced capacitance performance with a specific capacitance of 251 F g^−1^ at 0.5 A g^−1^, which compares favorably with the vast majority of two-dimensional COFs and other porous electrode materials.

## Abstract (web summary)

Covalent organic frameworks (COFs) are promising porous crystalline materials but controllable synthesis of COFs with uniform morphology remains challenging. Here, the authors report a self-templated synthesis of uniform and unique hollow spheres based on highly conjugated three-dimensional COFs.

## Introduction

Covalent organic frameworks (COFs), as an important type of crystalline porous materials with well-defined and predictable structures, demonstrate extensive applications in molecule storage and separation^1,2^, catalysis^3,4^, energy storage^5,6^, optoelectronics^7,8^, and sensors^9,10^. Despite their crystalline features, COFs are usually obtained as microcrystalline morphologies and their long-range ordered growth is limited in the nano/micro domain because of the internal defects and kinetic trapping of smaller crystallites^11–13^. The morphology of the crystallites is an important factor for their applications, such as molecular absorption and catalysis^14,15^, drug delivery^16,17^, energy storage^18,19^, etc. In general, the geometry of the building blocks plays a key role to determine the morphology of the crystallites^20–22^. Based on the geometry of the building blocks, COFs are mainly classified as two-dimensional (2D) COFs and three-dimensional (3D) COFs^23–25^. Since the first report on 2D COFs presented by Yaghi and co-workers in 2005 (ref. ^26^), extensive efforts have been devoted to this fast developing area^6,27,28^. Controllable synthesis of COF crystallites with uniform morphology, which is beneficial to fine modulating the material performance and better understanding the forming mechanisms of COF crystallites^29,30^, is highly desirable yet quite challenging^11,31,32^. Various COFs with uniform morphology such as free-standing films^33–36^, nanosheets^37–39^, tubes^12,40^, shuttle^41^, belts^22,42^, fibers^43,44^, and spheres^45,46^ have been recently reported mainly based on 2D COFs with fully exploring the potential of the 2D-layered structures. As compared with 2D COFs, 3D COFs with extended networks are generally able to achieve intrinsically large surface areas^47,48^, low density of open sites^49,50^, and fascinating confinement effects^51–53^, which have recently received increasing interest. However, the synthesis of 3D COFs with uniform morphologies remains unexplored. It is quite challenging to obtain 3D COF crystallite structures with uniform morphology mainly due to the “unavoidable” existence of cross-linked networks^53^. Considering their unique chemical and structural characteristics, it is charming to construct 3D COFs with uniform morphologies via controllable synthesis.

Herein, we report the controllable synthesis of 3D COFs (3D-Sp-COF) with uniform and unique hollow spherical morphology based on highly conjugated building blocks via self-templated synthetic methods. Their formation mechanism of hollow spheres based on 3D COFs has been investigated by a detailed time-dependent study and the growth process has been dominated by Ostwald ripening. Interestingly, all the 3D COFs show large surface area (up to 1016 m^2^ g^−1^) and high ion mobility, which serve as attractive candidates as electrode materials for energy storage applications. Among these, the well-defined hollow spherical 3D-Sp-COF@96 h shows enhanced specific capacitance (251 F g^−1^ at 0.5 A g^−1^) as compared with the 3D COFs with defective intermediate morphology, revealing that the characteristics of 3D COFs can be facilely regulated by exploring the potential of the microstructures. According to our preliminary specific capacitance studies, overwhelming high specific capacitance of 251 F g^−1^ has been achieved for 3D COFs by facilely modulating the morphological features.

## Results

### Synthesis and structural characterizations

**Fig. 1:**
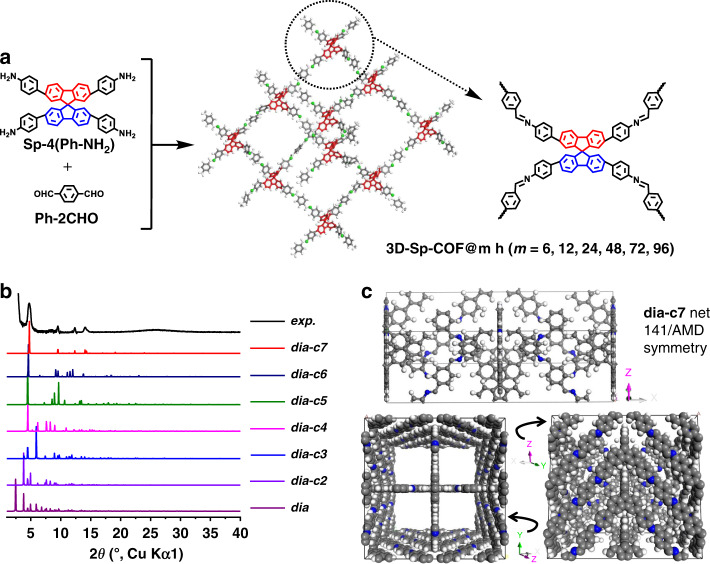
Synthesis and characterization of 3D-Sp-COFs. **a** Schematic illustration for the synthesis of 3D-Sp-COF (@*m* h is on behalf of the reaction time). **b** The experimental PXRD profiles of 3D-Sp-COF (black curve) compared with theoretical PXRD calculated from crystal models with different degrees of interpenetration. **c** Unit cell, top view, and side view of the cell of 3D-Sp-COF (dia-c7): C, gray; N, blue; H, white.

Schematic illustration for the synthesis of 3D-Sp-COF is depicted in Fig. 1a. Spirobifluorene core Sp-4(Ph-NH_2_) was synthesized by Suzuki cross-coupling reaction (Supplementary Figs. 1–4). 3D COF (3D-Sp-COF) with spirobifluorene core and imine bonds was prepared by the solvothermal reaction between Sp-4(Ph-NH_2_) and terephthalaldehyde (Ph-2CHO) with an acetic acid catalyst at 120 °C (Supplementary Fig. 5). For comparison, polymerization between Sp-4(Ph-NH_2_) and 2,5-dihydroxyterephthalaldehyde was also conducted under the same conditions to afford 3D-SpOH-COF (Supplementary Fig. 5). 3D-Sp-COF and 3D-SpOH-COF were isolated as yellow microgranular powder in 72% and orange powder in 68% yield, respectively. The crystalline nature of 3D-Sp-COF was determined by powder X-ray diffraction (PXRD) (Fig. 1b). As shown in the experimental PXRD pattern (Fig. 1b, black curve), 3D-Sp-COF exhibited intense diffraction peaks (2*θ*) at 4.73°, 9.52°, 12.36°, and 14.13° corresponding to the (200), (400), (411), and (501) Bragg planes of the space group I41/AMD, respectively, indicating high crystallinity of the resulting 3D COF. 3D-SpOH-COF exhibited intense diffraction peaks (2*θ*) at 4.17°, 8.46°, 10.07°, 12.76°, 13.92°, 19.78°, and 14.13° corresponding to the (200), (400), (211), (411), (431), and (631), respectively (Supplementary Figs. 6 and 7). Pawley refined PXRD patterns (Supplementary Fig. 6, red) with negligible difference (residuals *R*_p_ = 6.58%, *R*_wp_ = 12.18% for 3D-Sp-COF and residuals *R*_p_ = 6.53%, *R*_wp_ = 11.75% for 3D-SpOH-COF) compared favorably with the experimental patterns, which shows that the obtained crystal structure is very close to the predicted structure. To illustrate the lattice packing, the crystal models were constructed by employing Materials Studio 8.0 software package. Considering that 3D-Sp-COF was composed of tetragonal disphenoid-shaped spirobifluorene core and linear imine bonds, the possible lattice structures with different interpenetration degrees were proposed to demonstrate a fold-interpenetrated diamond (dia) topology similar to the reported SP-3D-COF 1 and SP-3D-COF 2 (ref. ^53^). According to the detailed simulation, the calculated PXRD pattern of 3D-Sp-COF (Fig. 1b, red curve) of sevenfold-interpenetrated diamond (dia-c7) net with I41/AMD symmetry was consistent with the experimental results. The refinement data of 3D-Sp-COF (*a* = *b* = 35.554 Å, *c* = 11.351 Å, *α* = *β* = *γ* = 90.0°) and 3D-SpOH-COF (*a* = *b* = 41.6510 Å, *c* = 9.9993 Å, *α* = *β* = *γ* = 90.0°, Fig. S5) both yielded a unit cell with parameters nearly equivalent to those of suggested models. The other possible lattice structures with non-, twofold-, threefold-, fourfold-, fivefold-, and sixfold-interpenetrated diamond topologies for 3D-Sp-COF were constructed with different space groups (Supplementary Figs. 8–14). However, the simulated PXRD patterns of these hypothetical topologies were not in agreement with the experimental results. Obviously, the lattice structures of 3D-Sp-COF and 3D-SpOH-COF (dia-c7) are different from those of SP-3D-COF 1 (dia-c6)^53^, which may play a role to affect the micro-morphology of the resulting COFs.

**Fig. 2:**
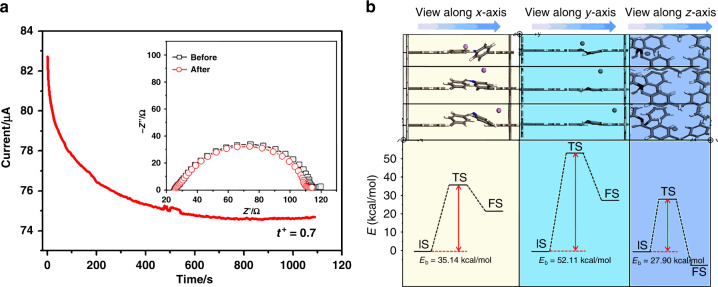
The lithium ion migration characterization of 3D-Sp-COF. **a** The chronoamperometry of the Li/3D-Sp-COF/Li symmetric cells (experiment condition: ambient temperature, potential step of 10 mV). The insets present the EIS before and after polarization at ambient temperature. **b** Theoretical calculation of Li-ion migration behaviors inside the pore (top) with corresponding energy diagrams (bottom). The initial, transition, and final states are abbreviated as IS, TS, and FS, respectively.

To elucidate the Schiff-base polymerization, Fourier transform infrared spectra of Sp-4(Ph-NH_2_), Ph-2CHO, and 3D-Sp-COF were tested (Supplementary Fig. 15). The aldehyde band at 1703 cm^−1^ and the N–H stretching band at 3372 cm^−1^ were significantly weakened after the Schiff-base reaction. However, the new C = N stretching band at 1625 cm^−1^ was significantly enhanced, which suggested the formation of the imine linkage in this 3D COF. Note that the C = O stretching band at 1703 cm^−1^ appeared in 3D-Sp-COF, probably due to moisture included in the pores. According to thermogravimetric analysis, 3D-Sp-COF showed an excellent thermal stability up to 475 °C (Supplementary Fig. 16). As shown in Fig. 2, 3D-Sp-COF is prone to transport ions (such as Li^+^) more efficiently. As predicted, the lithium-ion transference number (*t*^*+*^) of 3D-Sp-COF of 0.7 is recorded (Supplementary Table 1), which overwhelms most typical polyethylene oxide (PEO)-based polymer electrolytes^54,55^ and even comparable to the signal-ion electrolyte systems^56–58^. According to the results, ion conduction pathways provided by 3D COFs play a positive role on improving the ion transport. The lithium ion cell has a discharge capacity of 130, 120, and 75 mA h g^−1^ at the current densities of 54, 105, and 210 µA, respectively, suggesting superior cell performance using 3D-Sp-COF-based electrolyte (Supplementary Fig. 17). The lithium ion migration phenomenon of 3D-Sp-COF is explained by density functional theory calculation. Specifically, lithium ion migration in the pores is studied by comparing three pathways (*x*-axis pathway, *y*-axis pathway, and *z*-axis pathway; Supplementary Fig. 18). The results show that the Li-ion migration along the *x*-axis pathway and *z*-axis pathway require a lower energy barrier (Fig. 2b and Supplementary Table 2) as compared to that along the *y*-axis pathway. This preferred *x*- and *z*-axial Li-ion migration could be attributed to the shorter hopping distances, which is promoted by the N atoms of Schiff-base.

**Fig. 3:**
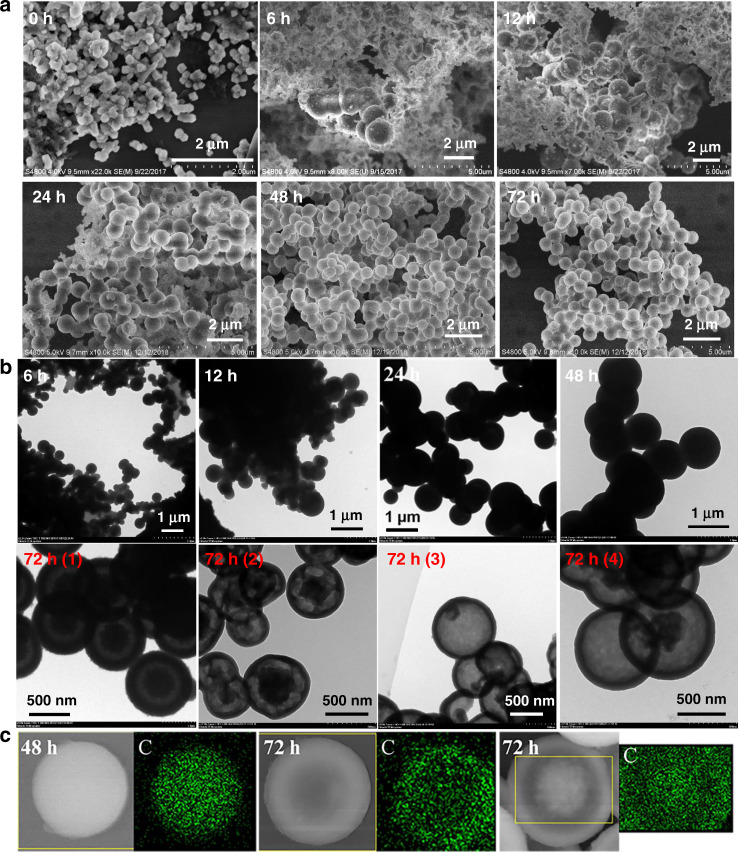
Structural characterization of 3D-Sp-COFs by different microscopic techniques. **a** FE-SEM images. **b** TEM images, 72 h (1)–(4) represents 3D-Sp-COF@72 h synthesized from different experimental batches. **c** SEM-energy-dispersive spectrum (EDS) elemental mapping images: solid sphere (left), full hollowing sphere (middle), and homogeneous core–shell sphere (right).

### Morphology characterizations

Interestingly, well-defined hollow spherical structures were observed for 3D-Sp-COF with an average diameter of 500–700 nm (Supplementary Fig. 19). To the best of our knowledge, this is the first hollow spheres based on 3D COFs. Similarly, 3D-SpOH-COF also forms well-defined spherical structure (Supplementary Fig. 20). Unlike hollow sphere structure of 3D-Sp-COF, 3D-SpOH-COF shows a solid sphere structure even with reaction time over 96 h (Supplementary Fig. 20b, 3D-SpOH-COF@96 h). In contrast, although SP-3D-COF 1 with similar spirobifluorene backbone structures shows the characteristics of crystallization, it exists in the form of irregular microcrystals^53^, whereas its long-range ordering growth in the nano/microdomain may have been limited due to the internal defects and kinetic trapping of smaller grains. The results suggest that the formation of spherical structure is closely related to chemical structures of the monomers, the reaction conditions, the type of unit cells, and the symmetry groups. To understand the formation mechanism of hollow spheres, the morphological changes at different reaction time (6, 12, 24, 48, 72, and 96 h) were studied by means of field emission scanning electron microscopy, transmission electron microscopy (TEM), and SEM-energy-dispersive spectrum (EDS) mapping (Fig. 3 and Supplementary Fig. 21). As shown in Fig. 3a, these nanospheres grew up gradually and formed uniform spherical structures with diameters of 500–700 nm as the reaction time increased. The rough surface on the wall of these nanospheres gradually became smooth. The size of the spheres increased obviously at the beginning of the reaction. After 24 h, the spheres size increased slowly and approximately keeps almost consistent with the increase of reaction time. It is important to note that the structure tend to be strong and stable only when the reaction time is long enough (>48 h). Otherwise, the structure will collapse during the washing process. As shown in Supplementary Fig. 22, the structure of 3D-Sp-COF@12 h was decomposed from nanopheres into irregular blocks after repeated washing. Furthermore, TEM images provided useful information to explore the growth pattern of these hollow spheres (Fig. 3b). It was found that 3D-Sp-COF@72 h from different experiment batches presented hollow spherical morphologies such as complete hollow spheres, symmetric core–shell hollow spheres, and asymmetric core–shell hollow spheres, which may originate from the packing of original nanoparticles and ripening characteristics. 3D-Sp-COF was also tested after soaking in different solvents for 2 h. Impressively, the crystal form and spherical structures of 3D-Sp-COF remained basically unchanged after treating by different solvents, which shows that 3D-Sp-COF has a good chemical stability (Supplementary Fig. 23). Various hollow spherical structures were also measured by SEM-EDS mapping (Fig. 3c). Based on these data, the construction process of these hollow spherical structures is similar to those of inorganic and organic hollow nanostructures, in which Ostwald ripening dominated the growth of spherical structures^59–61^.

### Mechanism of hollow sphere formation

**Fig. 4:**
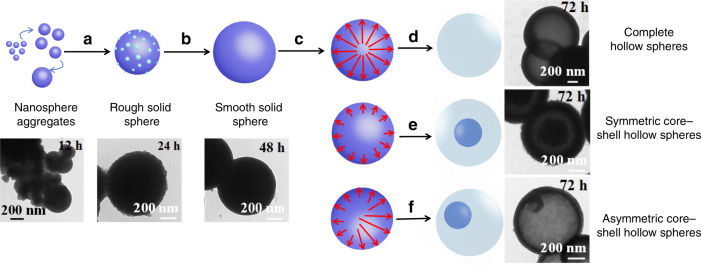
Self-templated hollowing evolvement process of 3D-Sp-COFs via Ostwald ripening mechanism. **a** Extinction of small particles and growth of large particles and continuous growth of large particles with dense cores and rough surfaces; **b** crystallites on the surface to achieve a smooth morphology; **c** dissolution and outmigration of inner crystallites; **d** formation of complete hollow spheres; **e** formation of symmetric core–shell hollow spheres; **f** formation of asymmetric core–shell hollow spheres.

Detailed Ostwald-ripening mechanism of hollow spheres based on 3D-Sp-COF is illustrated in Fig. 4. Firstly, due to the Schiff-base polymerization, a large number of small 3D COF microcrystals nucleate from solution and rapidly assemble into larger solid microspheres to reduce the surface energy. Subsequently, the size and density of the solid microspheres continue to grow. In the process of solid growth, the crystal structure on the spherical surface can facilitate the formation of nanoparticles outside the spheres to produce coarse microspheres (Fig. 4a). Next, the surfaces of these crystal microspheres gradually become smooth due to the presence of unreacted amino or aldehyde end functional groups (Fig. 4b). Meanwhile, the crystallization of 3D COF microspheres yielded hollow shell microspheres and microporosity via inside-out Ostwald-ripening mechanism (Fig. 4c). In this case, microspheres finally evolve into complete hollow spheres (Fig. 4d), symmetric core–shell spheres (Fig. 4e), and asymmetric core-shell spheres (Fig. 4f). As shown in Fig. 4, the complete hollow spheres evolve from the center of solid microspheres. The evacuation of symmetric core–shell spheres and asymmetric core–shell spheres begins at the surface of the solid particles^62^. Since the asymmetric Ostwald ripening occurs in the area with lower crystal density, the solid microspheres evolve into asymmetric core–shell spheres or more complex morphologies^63^.

### Gas adsorption

**Fig. 5:**
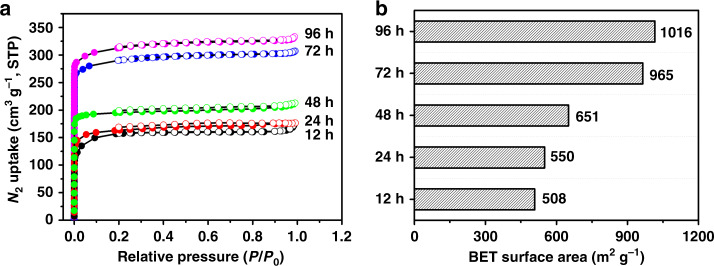
The porosity of 3D-Sp-COFs. **a** N_2_ adsorption–desorption isotherms of 3D-Sp-COF@*m* h (*m* = 12, 24, 48, 72, 96). **b** The Brunauer–Emmett–Teller **(**BET) surface area of 3D-Sp-COF-3@*m* h (*m* = 12, 24, 48, 72, 96).

The permanent porosity of 3D-Sp-COFs at various reaction time was characterized by the nitrogen adsorption–desorption isotherms at 77 K (Supplementary Figs. 24–28). As shown in Fig. 5a, all 3D-Sp-COFs showed a type I isotherm with significant increase under low relative pressure region (*P*/*P*_0_ < 0.05), demonstrating as a typical property of microporous molecule. The nonlocal density functional theory showed pore size distributions with a major pore width of 1.86 nm for 3D-Sp-COF@72 h and 3D-Sp-COF@96 h (Supplementary Fig. 29). What is more, the Brunauer–Emmett–Teller (BET) surface area of 3D-Sp-COFs increased with the increment of reaction time. The BET surface area were calculated as 508, 550, 651, 965, and 1016 m^2^ g^−1^ for 12, 24, 48, 72, and 96 h, respectively. Meanwhile, the total pore volume (*V*_P_) increased from 0.24 to 0.47 cm^3^ g^−1^ (*P*/*P*_0_ = 0.99). Based on these data, the specific surface area of hollow microspheres increased significantly compared with that of the solid microspheres.

### Electrochemical performance

**Fig. 6:**
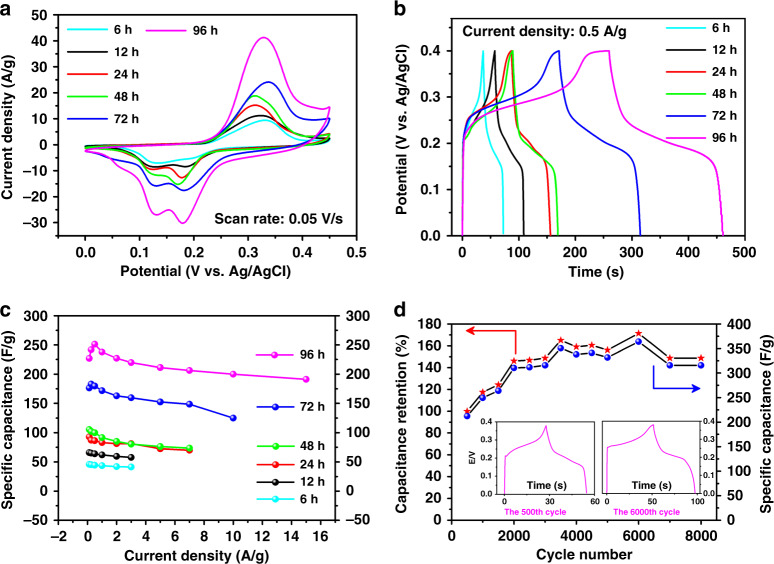
Electrochemical performance of 3D-Sp-COFs. **a** CVs at 50 mV s^−1^. **b** GCDs at 0.5 A g^−1^. **c** Specific capacitance dependence on current density. **d** Long-term cyclic stability performance of 3D-Sp-COF@96 h at 3 A g^−1^ in 6 M KOH electrolyte. Inset: GCD curves of the 500th and the 6000th cycle, respectively.

Inspired by our previous work^64–66^, the large surface area and conjugated microporous property of 3D-Sp-COF**s** suggest potential applications in supercapacitance. Cyclic voltammetry (CV) and galvanostatic charge–discharge (GCD) experiments for 3D-Sp-COFs were performed to explore the electrochemical characteristics (Supplementary Figs. 30–35). The reversible redox peaks in the CV plots indicate the specific capacitance is pseudocapacitive in nature. The area under the CV curve of 3D-Sp-COF@96 h with well-defined hollow spherical structure is significantly larger than that of 3D-Sp-COF@6 h for nanoparticles, indicating that the uniform hollow spherical structures are beneficial to improving the capacitance performance (Fig. 6a). It was further verified by GCD test (Fig. 6b). The capacitance performance increases with the increment of reaction time, matching well with CV data mentioned above. The calculated specific capacitances for 3D-Sp-COF@6 h, 3D-Sp-COF@12 h, 3D-Sp-COF@24 h, 3D-Sp-COF@48 h, 3D-Sp-COF@72 h, and 3D-Sp-COF@96 h are 45, 64, 86, 100, 180, and 251 F g^−1^, respectively, at current densities of 0.5 A g^−1^. Most notably, the specific capacitance of uniform hollow spheres 3D-Sp-COF@96 h is more than twice that of the solid spheres 3D-Sp-COF@48 h. As far as we are concerned, the excellent capacitive value of hollow spheres based on 3D-Sp-COF is superior to that of 2D COF-based materials previously reported (Supplementary Table 3). Moreover, the capacitance performance overwhelms those of porous electrode molecules, including metal organic frameworks, conjugated microporous polymers, porous triazine-based frameworks, and N-doped carbon nanotubes (Supplementary Table 4)^67–69^. In order to study the capacitance stability at high power density, the specific capacitances at different current densities (0.125–15 A g^−1^) were tested (Fig. 6c). The specific capacitance of 3D-Sp-COF@96 h maintained above 191 F g^−1^ at a high current density of 15 A g^−1^, implying that such excellent capacitance behaviors can still remained at high power density due to the stable hollow spherical structures. Subsequently, the cycling stability of 3D-Sp-COF@96 h was investigated at current density of 3 A g^−1^ (Fig. 6d). Unexpectedly, the specific capacitance increased dramatically with the increase of cycle numbers, reaching a maximum of 364 F g^−1^ at the 6000th cycle. Even after 8000 cycles, the specific capacitance remained at 316 F g^−1^, which was 1.4 times as large as the initial value. The increase of specific capacitance during cyclic scanning may be ascribed to the wetting activation of the hollow spheres, which leads to the effective transport of electrolyte ions in the ordered channels. The electron and ion transport characteristics of the 3D-Sp-COF@96 h were further studied by electrochemical impedance spectroscopy (Supplementary Fig. 36). The Nyquist curve shows a steep straight line in the low-frequency region with a small intercept, indicating ideal capacitance behaviors. In addition, there is almost no semicircle in the high frequency region, suggesting that its conductivity is relatively ideal.

## Discussion

In summary, a set of highly conjugated 3D-Sp-COFs was designed and synthesized, beginning from tetrahedral tetraamine and terephthalaldehyde with spirobifluorene core structure as building blocks by means of imine condensation reactions. PXRD patterns and modeling studies demonstrated these 3D COFs are seven-fold-interpenetrated microporous diamond (dia-c7) net with I41/AMD symmetry. Remarkably, 3D-Sp-COF crystallites self-assembled into uniform hollow microspheres with narrow size distribution (500–700 nm) and large surface area (1016 m^2^ g^−1^), which were proved by SEM, TEM, EDS, and BET investigations. The mechanism of hollow spheres formation was studied using a detailed real-time tracking method, indicating that the formation of hollow spheres is the result of Ostwald ripening mechanism. In the Li/3D-COF/Li symmetric cells, ion conduction pathways provided by 3D COF are beneficial for enhancing the ion transport and the *t*^*+*^ of 3D-Sp-COF approaches 0.7. The value overwhelms most of the typical PEO-based polymer electrolytes and even comparable to the signal-ion electrolyte systems. Because of the large surface area and hollow spherical architecture, the 3D COF shows a high specific capacitance of 251 F g^−1^ at a current density of 0.5 A g^−1^, which is superior to the vast majority of 2D COFs and other porous electrode materials. Remarkably, the specific capacitance increases dramatically with the numbers of cycles, achieving a maximum of 364 F g^−1^ at the 6000th cycle due to the wetting activation of the hollow spheres. This study provides a feasible morphological modulation strategy to improve the electrochemical characteristics, which provides a deep insight into the design and synthesis of efficient and stable electrode materials for energy storage applications.

## Methods

### Materials

2,2′,7,7′-Tetrabromo-9,9′-spirobifluorene, 4-aminophenylboronic acid pinacol ester, terephthalaldehyde, and 2,5-dihydroxyterephthalaldehyde were purchased from Energy Chemical. All other reagents and solvents were commercially available and used as received.

### Synthesis of 3D-Sp-COF

A Pyrex tube was charged with 4,4′,4″,4‴-(9,9′-spirobifluorene-2,2′,7,7′-tetrayl)tetraaniline (68.1 mg, 0.1 mmol), terephthalaldehyde (26.8 mg, 0.2 mmol), and solvent mixture of 1,2-dichlorobenzene/*n*-butanol/6 M aqueous acetic acid (7:3:1, by volume). This mixture was sonicated for 5 min in order to get a homogeneous dispersion. The tube was then flash frozen at 77 K (liquid N_2_ bath), degassed by freeze–pump–thaw technique for three times. The tube was sealed off and then placed in an oven at 120 °C for a certain period of time (6, 12, 24, 48, 72, 96 h). The resulting precipitate was filtered, washed with anhydrous tetrahydrofuran, and extracted by Soxhlet extractor for 24 h. The solid was dried at 80 °C under vacuum for 12 h to afford yellow powder (63.0 mg, 72% yield).

### Synthesis of 3D-SpOH-COF

A Pyrex tube was charged with 4,4′,4″,4‴-(9,9′-spirobifluorene-2,2′,7,7′-tetrayl)tetraaniline (68.1 mg, 0.1 mmol), 2,5-dihydroxyterephthalaldehyde (33.2 mg, 0.2 mmol), and solvent mixture of 1,2-dichlorobenzene/*n*-butanol/6 M aqueous acetic acid (8:5:1, by volume). This mixture was sonicated for 5 min in order to get a homogeneous dispersion. The tube was then flash frozen at 77 K (liquid N_2_ bath), degassed by a freeze–pump–thaw technique for three times. The tube was sealed off and then placed in an oven at 120 °C for a certain period of time (12, 24, 48, 72, 96 h). The resulting precipitate was filtered, washed with anhydrous tetrahydrofuran, and extracted by a Soxhlet extractor for 24 h. The solid was dried at 80 °C under vacuum for 12 h to afford orange powder (63.9 mg, 68% yield).

## Supplementary information

Supplementary Information

## Data Availability

All data needed to evaluate the conclusions given in the paper are present in the Article and Supplementary Information. Any additional data related to this paper may be requested from the corresponding authors.
