# Supplementary material for: Self-templated synthesis of uniform hollow spheres based on highly conjugated three-dimensional covalent organic frameworks
Source: Nat Commun. 2020 Nov 3;11:5561. doi: 10.1038/s41467-020-18844-4 (PMC7642269; doi:10.1038/s41467-020-18844-4)
Supplement: Supplementary file 1 — Supplementary Information [file 41467_2020_18844_MOESM1_ESM.pdf]

Supplementary Information

Self-Templated Synthesis of Uniform Hollow Spheres Based on Highly Conjugated Three-Dimensional Covalent Organic Frameworks

Liu et al.

1. Supplementary Methods

Nuclear magnetic resonance (NMR) spectroscopy. NMR spectra were recorded on a Bruker Ultra Shield Plus 400 MHz NMR (^{1}H: 400 MHz, ^{13}C: 101 MHz).

Matrix Assisted Laser Desorption Ionization-time of Flight (MALDI-TOF) Mass Spectrometry. MALDI-TOF mass spectrometry analysis was performed on a Shimadzu AXIMA-CFR mass spectrometer in positive ion, reflector mode.

Fourier transform infrared (FT-IR) spectroscopy. FT-IR spectra were recorded on a L1600400 Spectrum TWO DTGS.

Powder X-ray diffraction (PXRD) analysis. PXRD patterns were carried out with a Bruker D8 Advance diffractometer with Cu K_{α} line focused radiation at 40 kV and 40 mA within a 2θ range from 1 to 40° with a step size of 0.02°. The powdered sample was added to the silicon wafer and compacted for measurements.

Thermogravimetric analysis (TGA). TGA from 20~800°C was carried out on NETZSCH STA 2500 in nitrogen atmosphere using a 10°C min^{-1} ramp without equilibration delay.

Scanning electron microscopy (SEM). SEM (Hitachi S4800) was used to investigate the structure of the as-prepared 3D-Sp-COF at an accelerating voltage of 4.0~5.0 kV. Samples were prepared by first dispersing the materials into ethanol or DCM assisted by ultrasonication. A drop of as-prepared solution was cast onto a silicon wafer and then attached to a flat aluminum sample holder.

Transmission electron microscopy (TEM). TEM (JEOL JEM-3010x) was used to investigate the structure of the as-prepared 3D-Sp-COF at an accelerating voltage of 100 kV. TEM samples were prepared by first dispersing the materials into ethanol or DCM assisted by ultrasonication. A drop of as-prepared solution was cast onto a holey carbon film of a copper TEM grid for examination.

Nitrogen isotherm measurements. The pore structure of 3D-Sp-COF sample was assessed from the N_2 isotherm curve measured by a gas adsorption analyzer (V-Sorb 2800P, Gold APP Corp., Beijing, China). Prior to the adsorption experiments, all samples were degassed at 150°C for 3 h to eliminate the surface contaminants (water or oil).

Preparation procedures of the work electrodes and electrochemical measurements. Electrochemical studies on the as-prepared electrodes were carried out on a CHI 660D electrochemical working station (Shanghai Chenhua Instrument, Inc.). The capacitor performances of 3D-Sp-COF were studied in 6 M KOH aqueous electrolyte solution using a standard three-electrode system, in which an Ag/AgCl (saturated KCl) electrode and a platinum wire were used as the reference and counter electrodes, respectively. The working electrode was fabricated as followed. 85 wt% 3D-Sp-COF material, 10 wt% carbon black, 5 wt% poly-tetrafluoroethylene (PTFE) as the binder and an appropriate amount of ethanol were mixed thoroughly to make slurry and then were coated onto a Ni foam (1×1 cm^2). The working electrode was dried in an oven at 80°C for a day. Cyclic voltammetry (CV) measurements were performed in the potential window of 0~0.45 V versus Ag/AgCl at various scan rates from 5 mV s^{-1} to 100 mV s^{-1}. The Galvanostatic charge/discharge (GCD) experiments were measured at different current densities ranging from 0.125 A g^{-1} to 15 A g^{-1}. The specific capacitances were calculated from the charge/discharge potential-time curve using the equation $C=i\Delta t/\Delta V$, where C (F g^{-1}) was specific capacitance, i (A g^{-1}) represents charge/discharge current density, Δt (s) was the total discharge time, and ΔV (V) was potential window. For the electrochemical impedance spectroscopy (EIS) analysis the frequency was varied from higher frequency of 10^5 Hz to a lower frequency of 0.01 Hz at open circuit potential with an amplitude of 10 mV. 5 μL butanedinitrile/30 wt% LiTFSI were added on the surface of the 3D COF pellet as the electrolyte to test the lithium-ion transference number (t^+). The lithium-ion battery was prepared

using the 3D-Sp-COF as electrolyte, while $LiCoO_2$-based material and Li metal as cathode and anode, respectively. The weight ratio of $LiCoO_2$, super P and PVDF was 90:5:5 for $LiCoO_2$-based cathode. Moreover, the cell performance evaluation of the cell was conducted on a LANHE CT2001A battery testing system.

Density-functional theory (DFT) calculations. All the DFT calculations were performed using the Dmol^3 software package based on the linear combination of atomic orbitals (LCAO) method. Electron-ion interactions were described using the DFT Semi-core Pseudopots (DSPP) pseudopotentials. A double numerical polarized (DNP) basis set was employed to expand the wave functions with an orbital cutoff of 3.3 Å for O, 3.1 Å for H, 3.7 Å for C, 3.4 Å for N 5.1 Å for Li. For the electron-electron exchange and correlation interactions, the functional parametrized by Perdew-Burke-Ernzerhof (PBE), a form of the general gradient approximation (GGA), was used throughout. The vander Waals interaction was described using the DFT-D2 method that proposed by Grimme. During the geometry optimizations, the atoms far from the reaction center were fixed at the bulk position. The convergence criterion for the electronic self-consistent field (SCF) loop was set to 10^{-5}. The atomic structures were optimized until the residual forces were below 0.004 Ha Å^{-1}. The transition state search was conducted via the quadratic synchronous transit (QST) method, and the tolerance for RMS of force was set to 0.004 Ha Å^{-1}. When lithium ions migrate in crystal materials, the conductivity is $\delta = nq\mu = A_S \exp(-\frac{W_S}{kT})$, where μ is ion mobility, A_s is proportional coefficient, W_s is conductivity activation energy, k is Boltzmann constant, and T is absolute temperature. For lithium ions, the diffusion coefficient is D$= a^2 v * \exp(-\frac{E_b}{kT})$, where a is the distance of migration jump, v is jump frequency (~10^{13} s^{-1}), E_b is migration energy of barrier, k is Boltzmann constant, and T is absolute temperature. The relationship between D and μ is μ= D $* \frac{q}{kT}$. For lithium-ion batteries, the conductivity is $\delta = \mathrm{n}q^2 D/kT$, where n is the number of lithium ions per unit volume.

Structural simulation and PXRD analysis. Crystal models for 3D-Sp-COF and 3D-SpOH-COF were established by Materials Studio (Ver. 8.0) suite of programs. Vertex positions were pre-set according to Reticular Chemistry Structure Resource (RCSR) with the dia net as reference. Subsequently, the real unit cell and symmetry were revealed by "finding symmetry". Geometry optimization of the established models was performed by Materials Studio Forcite Module (Universal force fields, Ewald summations). Optimized cell parameters were obtained at the same time. Then the computed PXRD profiles were simulated from the structure models using the Materials Studio Reflex Module. Possible structures with different degrees of interpenetration were tested in comparison with the experimental Powder X-ray diffraction (PXRD) data. Minor correction of cell parameters followed by repetitive geometry optimization was also employed for detailed comparison with experimental PXRD profiles. For 3D-Sp-COF and 3D-SpOH-COF, a 7-fold-interpenetrated diamond (dia-c7) net was identified as the most possible structure after detailed comparison.

Synthesis of Sp-4(Ph-NH$_2$). A mixture of 2,2',7,7'-tetrabromo-9,9'-spirobifluorene (1264 mg, 2.0 mmol), 4-aminophenyl-boronic acid pinacol ester (2191 mg, 10.0 mmol), tetrabutylammonium bromide (TBAB) (345 mg, 10 wt%), K_2CO_3 (aq., 2 M) (20 mL) and toluene (40 mL) was carefully degassed before and after $Pd(PPh_3)_4$ was added. The mixture was stirred and refluxed under nitrogen for 72 h. After being cooled to room temperature, most of the toluene was removed by rotary evaporator. Then, the mixture was poured into 100 mL water and extracted with 100 mL ethyl acetate (EA) for three times. After evaporated the solvent of combined organic layers, the crude product was purified by Alumina-B column chromatography using PE/EA (1:4) as eluent to get grayish-white solid. By recrystallization from *n*-hexane finally, white powder of 4,4',4'',4'''-(9,9'-spirobifluorene-2,2',7,7'-tetrayl)tetraaniline (Sp-4(Ph-NH$_2$)) was obtained in ~72% yield (980 mg).

2. Supplementary Figures

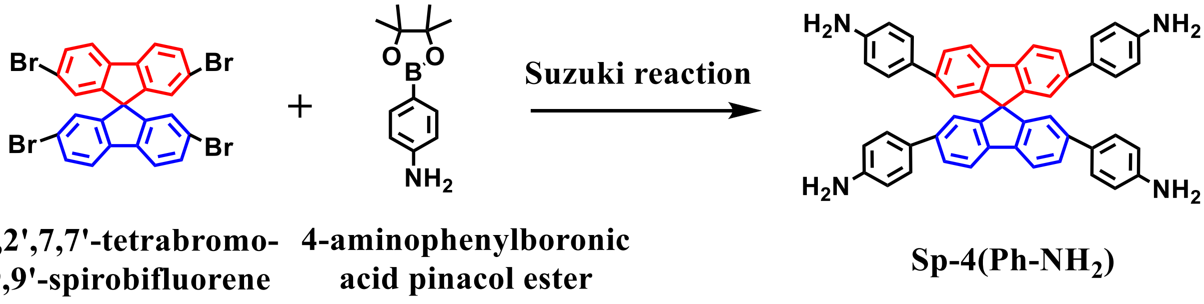

Supplementary Figure 1. Synthesis of 4,4',4'',4'''-(9,9'-spirobifluorene-2,2',7,7'-tetrayl)tetraaniline

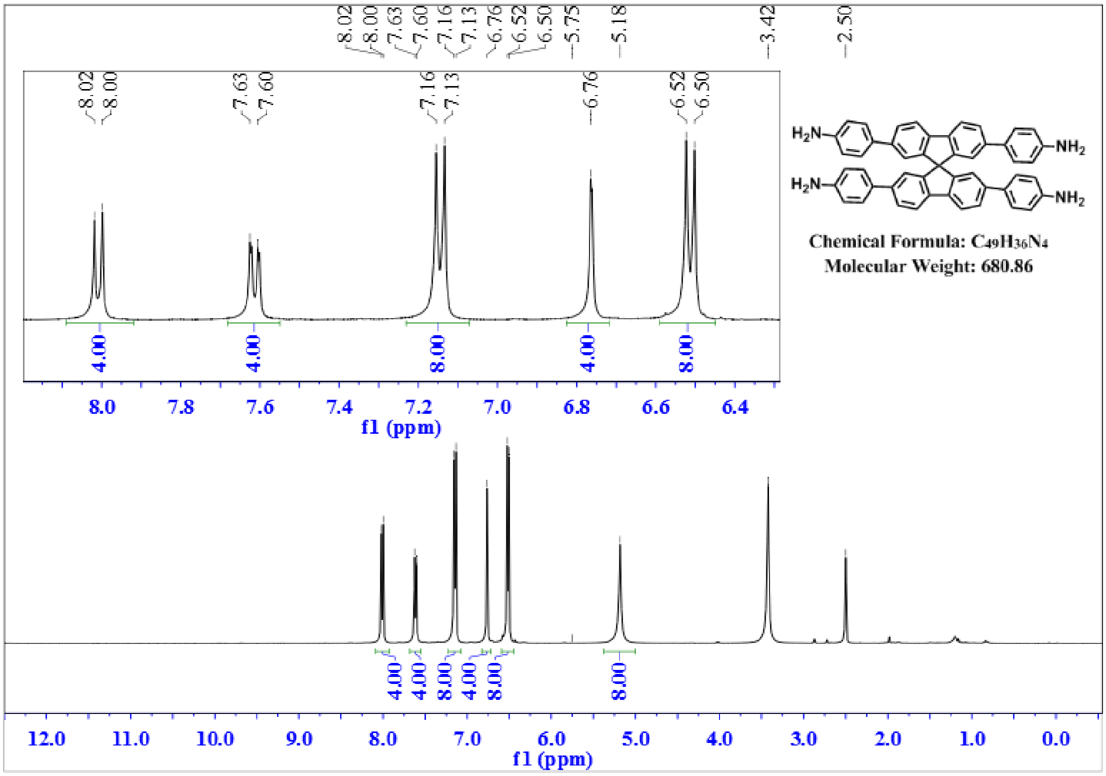

Supplementary Figure 2. ^{1}H-NMR for 4,4',4'',4'''-(9,9'-spirobifluorene-2,2',7,7'-tetrayl)tetraaniline.

Inset showing the zoom portion.

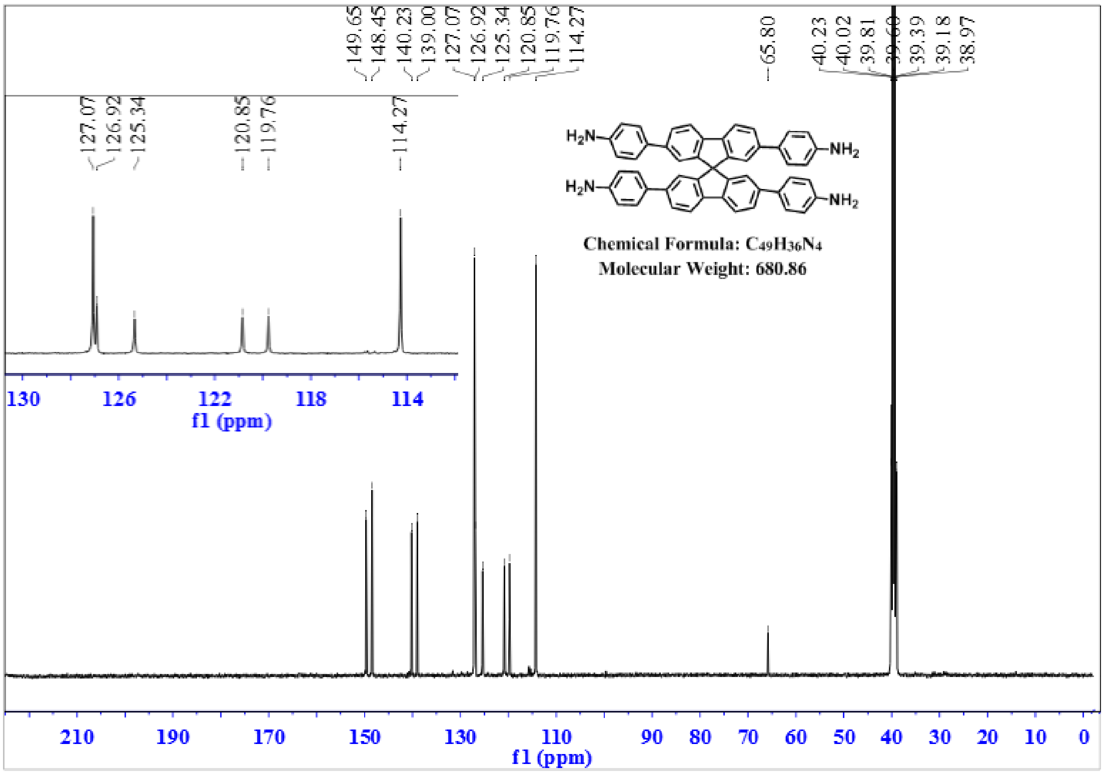

Supplementary Figure 3. ^{13}C-NMR for 4,4',4'',4'''-(9,9'-spirobifluorene-2,2',7,7'-tetrayl)tetraaniline.

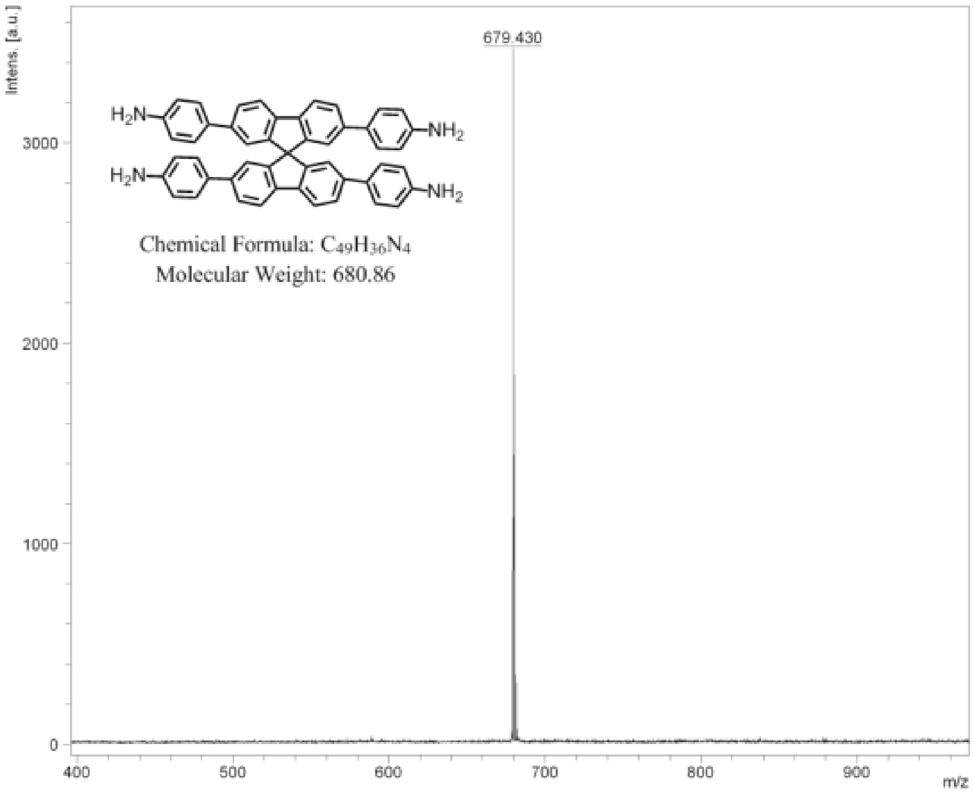

Supplementary Figure 4. MALDI-TOF for 4,4',4'',4'''-(9,9'-spirobifluorene-2,2',7,7'-tetrayl) tetraaniline.

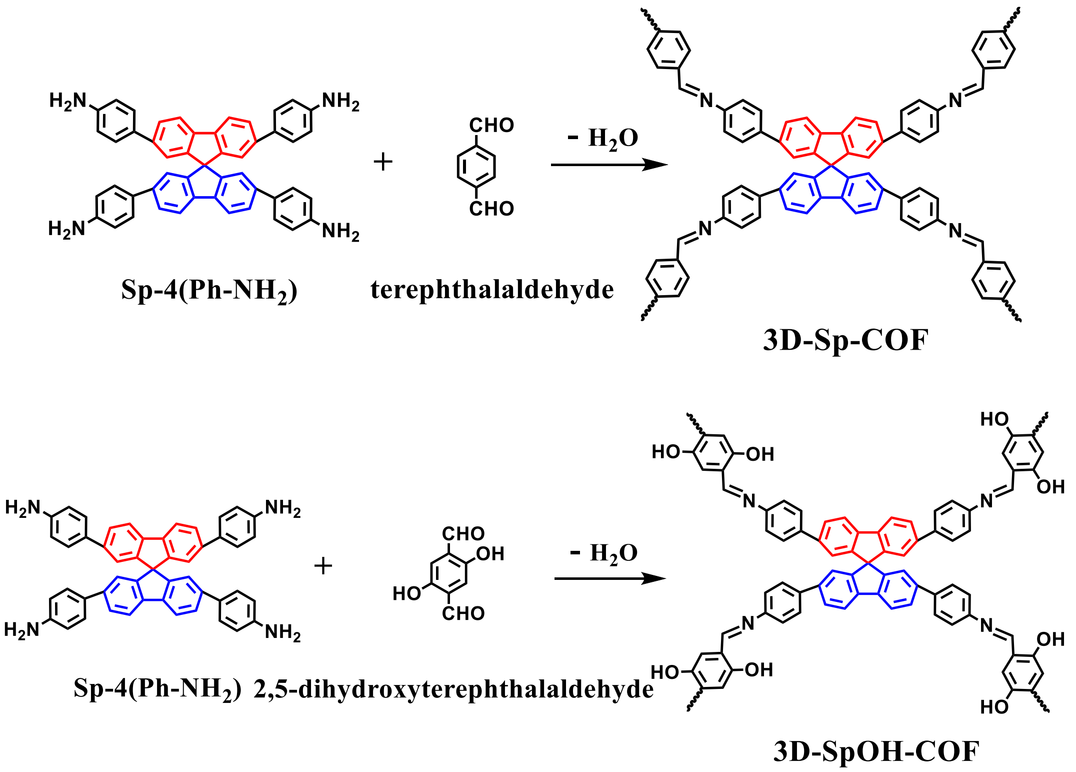

Supplementary Figure 5. Synthesis of 3D-Sp-COF and 3D-SpOH-COF.

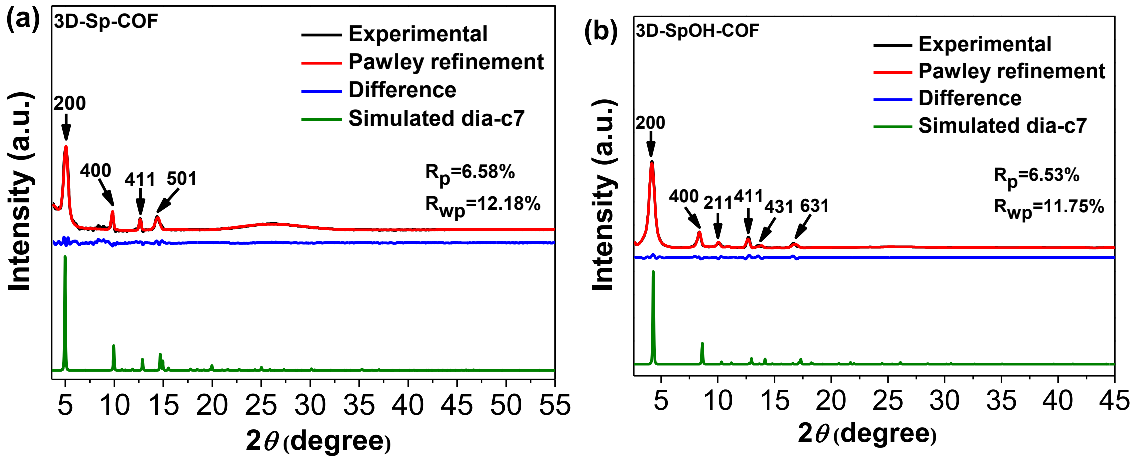

Supplementary Figure 6. The experimental (black), Pawley refined (red), the calculated PXRD patterns (green) from dia-c7 net of 3D-Sp-COF (a) and 3D-SpOH-COF (b) the difference between the experimental and refined PXRD patterns (blue).

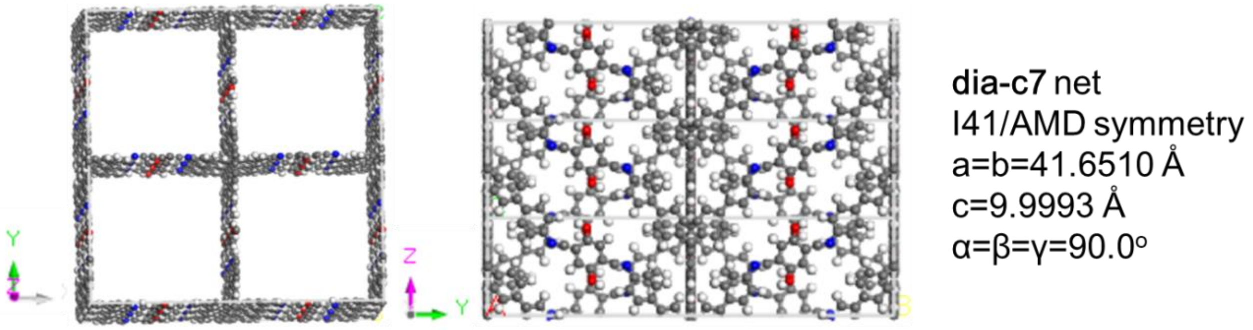

Supplementary Figure 7. Unit cell, top view and side view of the cell of 3D-SpOH-COF (dia-c7): C, gray; O, red; N, blue; H, white.

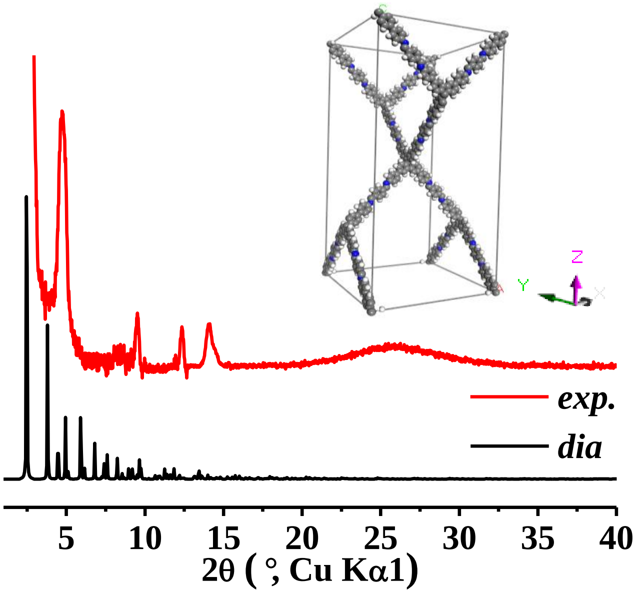

Supplementary Figure 8. The experimental PXRD curve (red) and theoretical PXRD curve basing on the non-interpenetrated dia topology (black) for 3D-Sp-COF. Inset: schematic representation of unit cell, C, gray; N, blue; H, white.

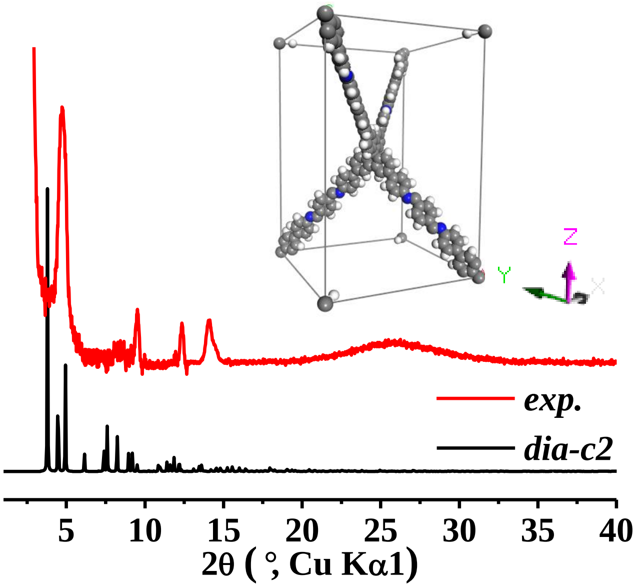

Supplementary Figure 9. The experimental PXRD curve (red) and theoretical PXRD curve based on the 2-fold-interpenetrated dia topology (black) for 3D-Sp-COF. Inset: schematic representation of unit cell, C, gray; N, blue; H, white.

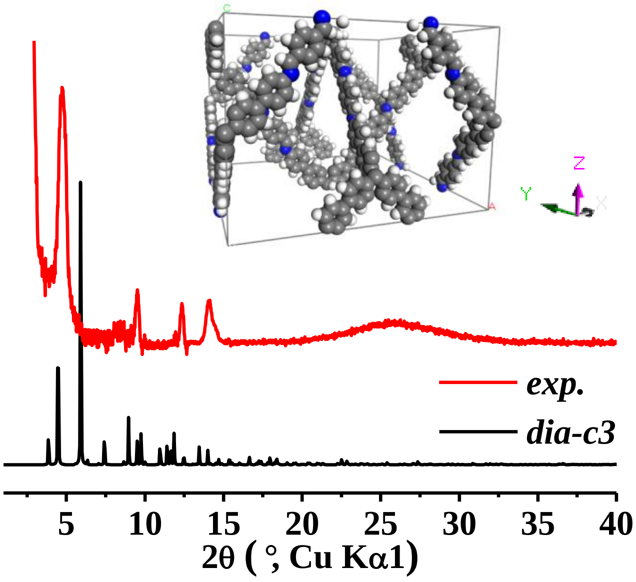

Supplementary Figure 10. The experimental PXRD curve (red) and theoretical PXRD curve based on the 3-fold-interpenetrated dia topology (black) for 3D-Sp-COF. Inset: schematic representation of unit cell, C, gray; N, blue; H, white.

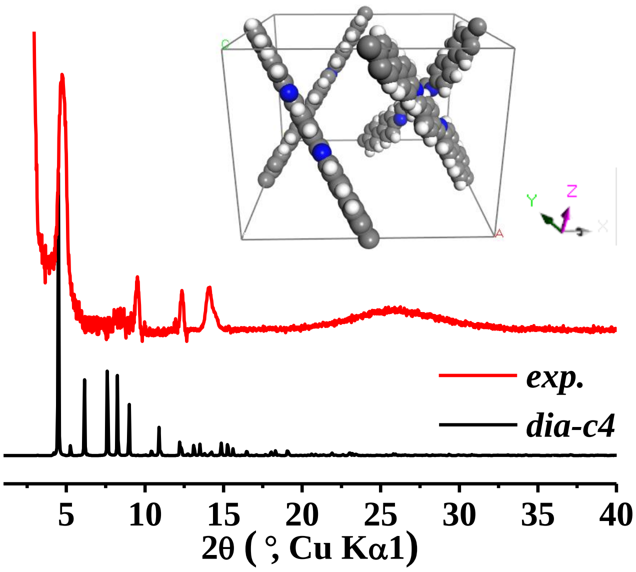

Supplementary Figure 11. The experimental PXRD curve (red) and theoretical PXRD curve based on the 4-fold-interpenetrated dia topology (black) for 3D-Sp-COF. Inset: schematic representation of unit cell, C, gray; N, blue; H, white.

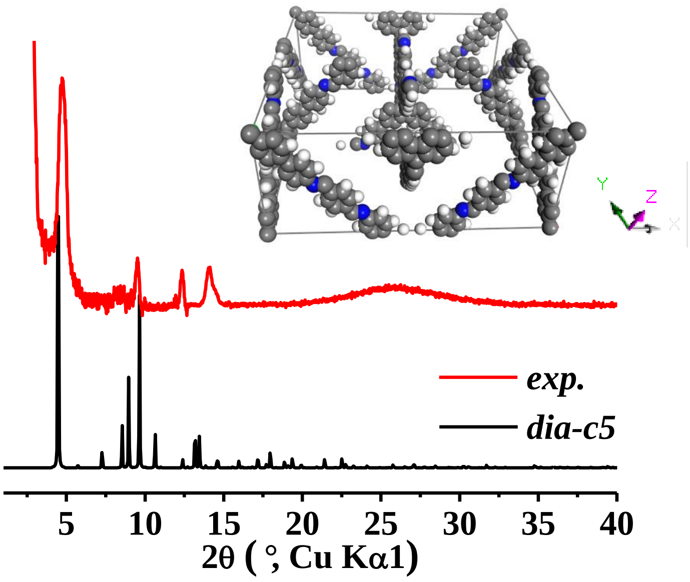

Supplementary Figure 12. The experimental PXRD curve (red) and theoretical PXRD curve based on the 5-fold-interpenetrated dia topology (black) for 3D-Sp-COF. Inset: schematic representation of unit cell, C, gray; N, blue; H, white.

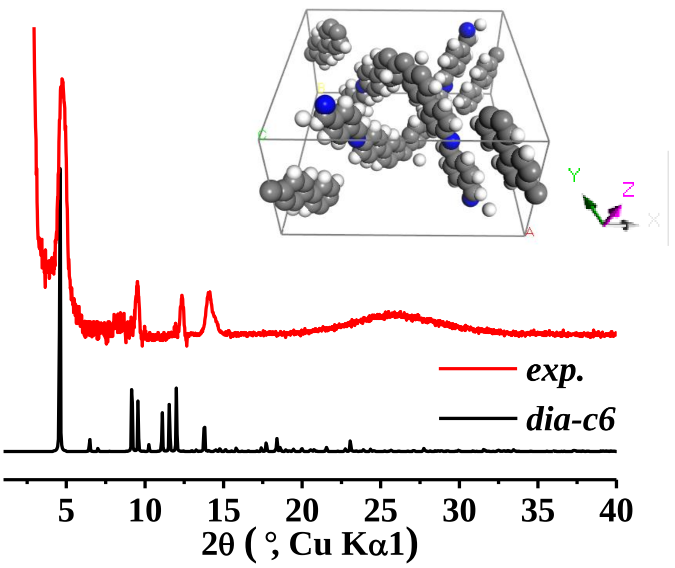

Supplementary Figure 13. The experimental PXRD curve (red) and theoretical PXRD curve based on the 6-fold-interpenetrated dia topology (black) for 3D-Sp-COF. Inset: schematic representation of unit cell, C, gray; N, blue; H, white.

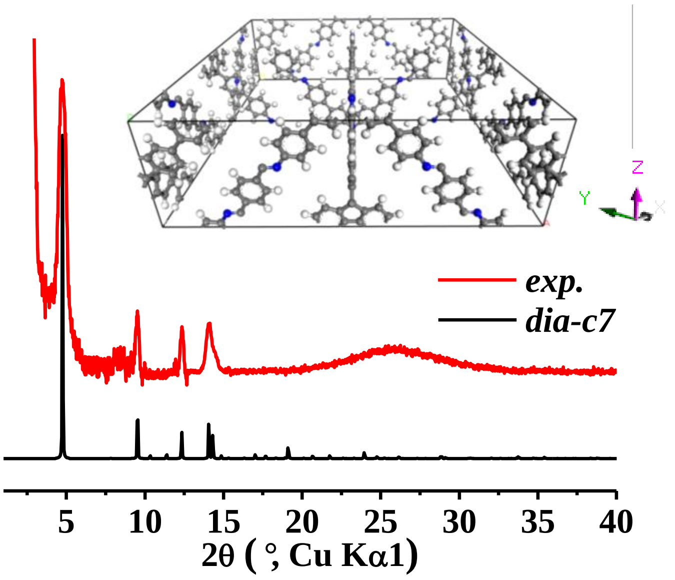

Supplementary Figure 14. The experimental PXRD curve (red) and theoretical PXRD curve based on the 7-fold-interpenetrated dia topology (black) for 3D-Sp-COF. Inset: schematic representation of unit cell, C, gray; N, blue; H, white.

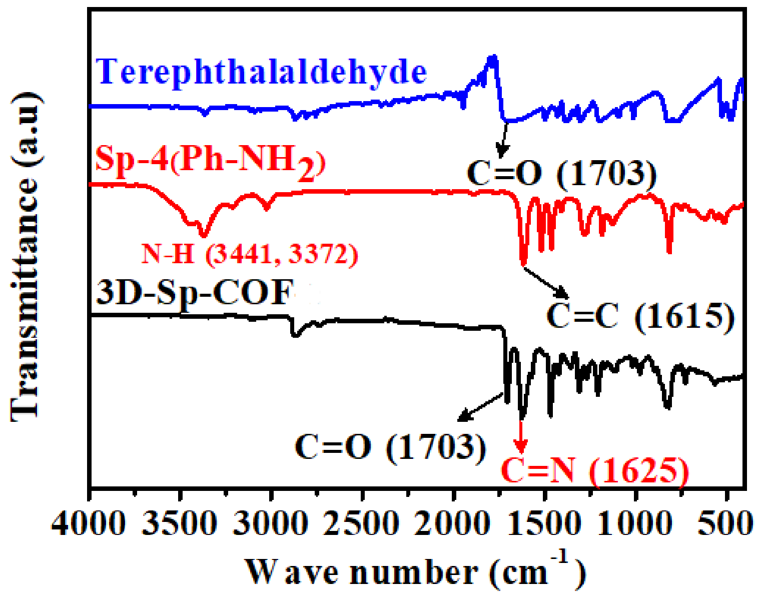

Supplementary Figure 15. FT-IR spectra of terephthalaldehyde monomer (Ph-2CHO, blue), Sp-4(Ph-NH_2) (red) and 3D-Sp-COF (black).

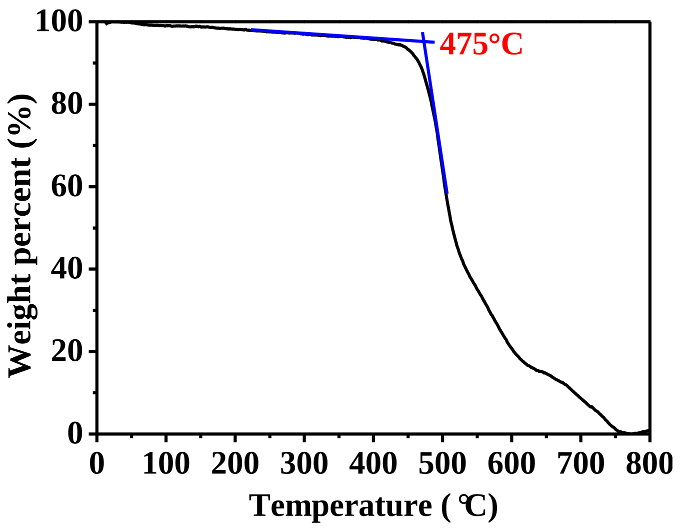

Supplementary Figure 16. TGA curve of 3D-Sp-COF.

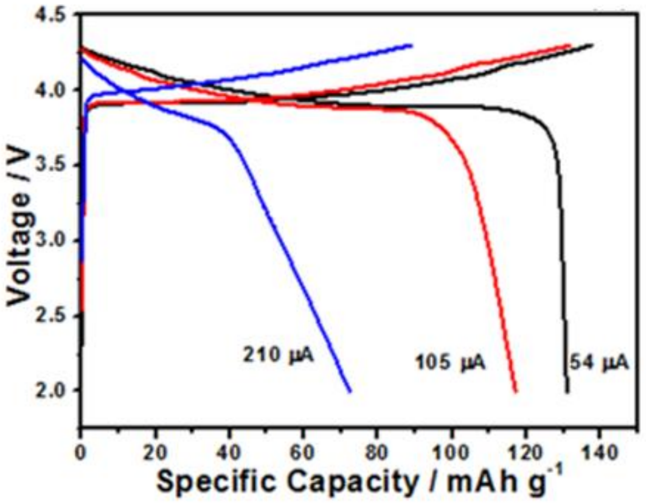

Supplementary Figure 17. Charge-discharge curves of the $LiCoO_2$/3D-Sp-COF-based electrolyte/Li cell at different current densities (tested at room temperature).

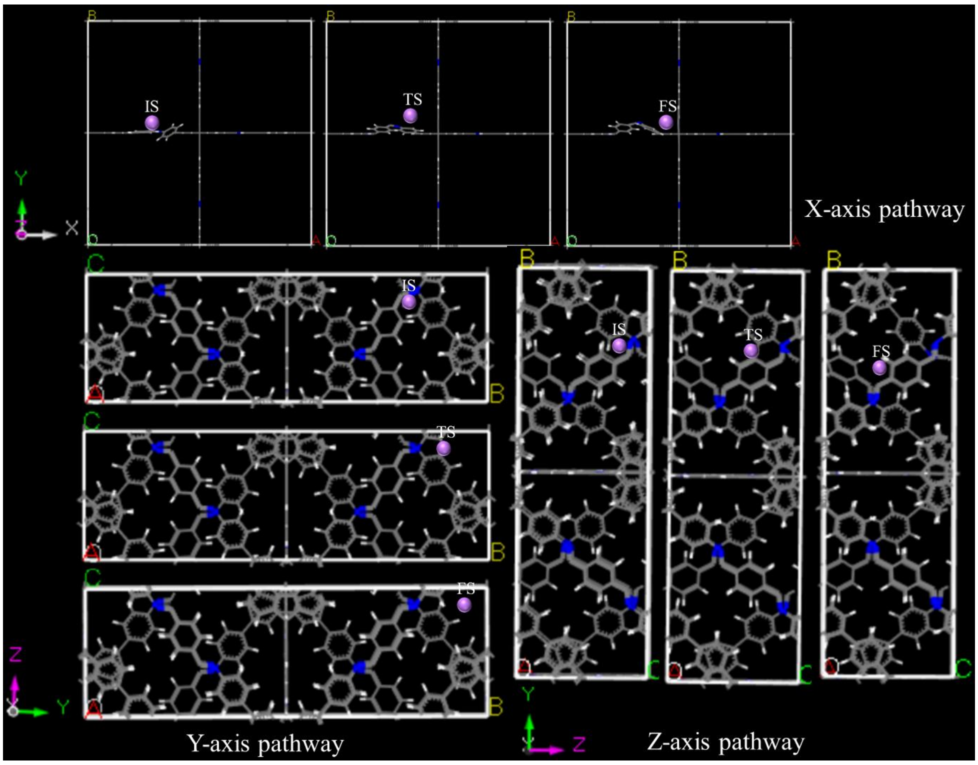

Supplementary Figure 18. Li-ion migration along the x-axis pathway, y-axis pathway, and z-axis pathway.

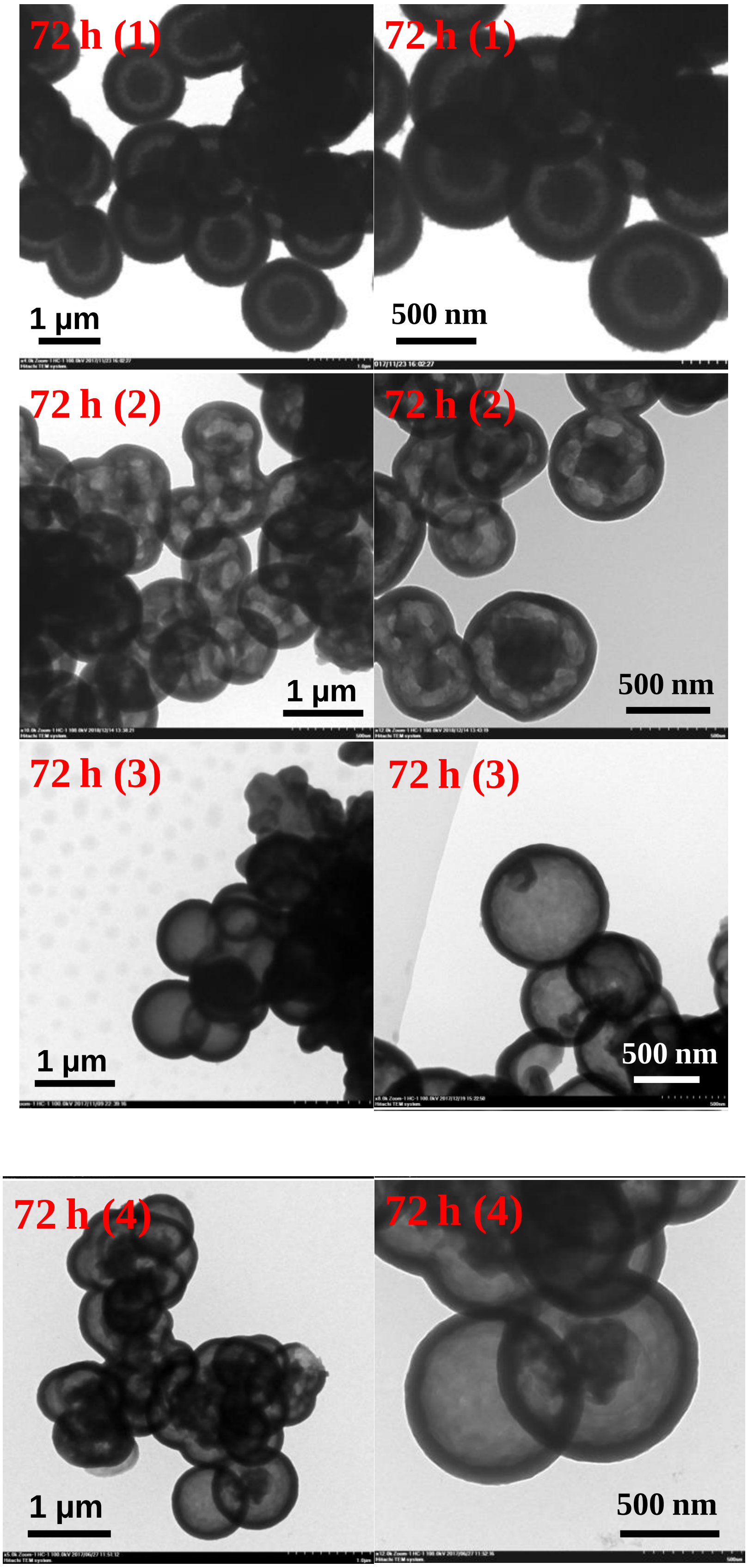

Supplementary Figure 19. TEM images of 3D-Sp-COF@72 h. Label 72 h (1)-(4) represents 3D-Sp-COF synthesized from different experimental batches with reaction time of 72 h.

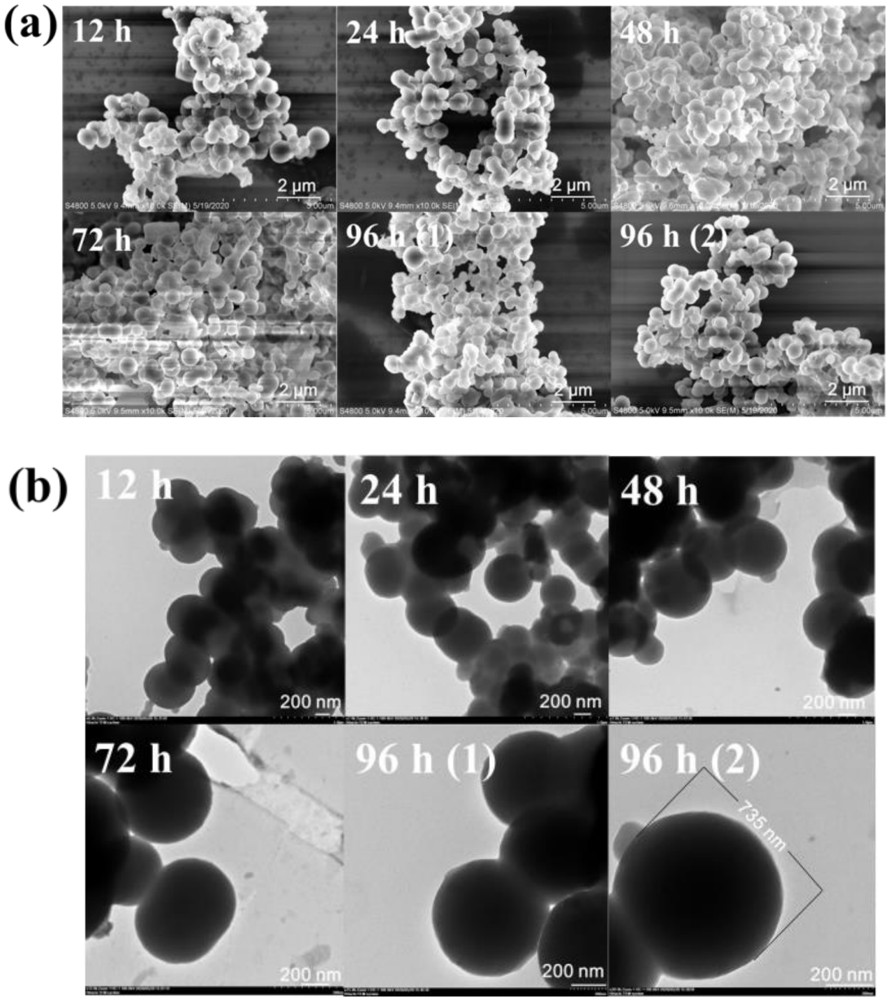

Supplementary Figure 20. SEM images (a) and TEM images (b) of 3D-SpOH-COF@m h (m=12, 24, 48, 72, 96), 96 h (1)-(2) represents 3D-SpOH-COF@96 h synthesized from different experimental batches.

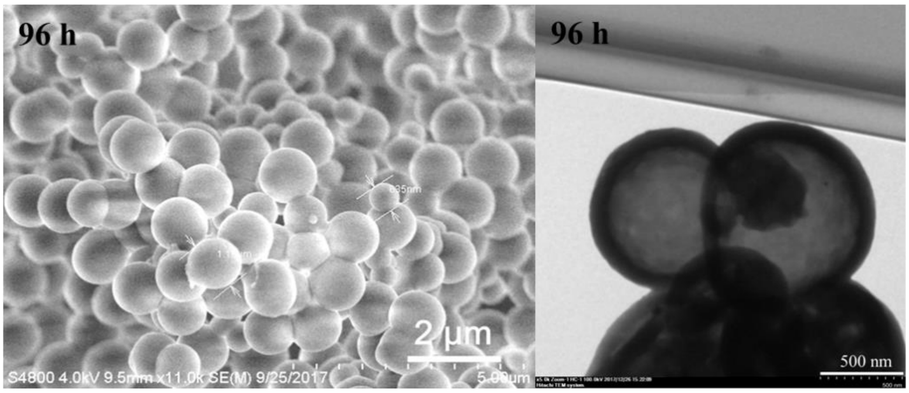

Supplementary Figure 21. SEM and TEM images of 3D-Sp-COF@96 h.

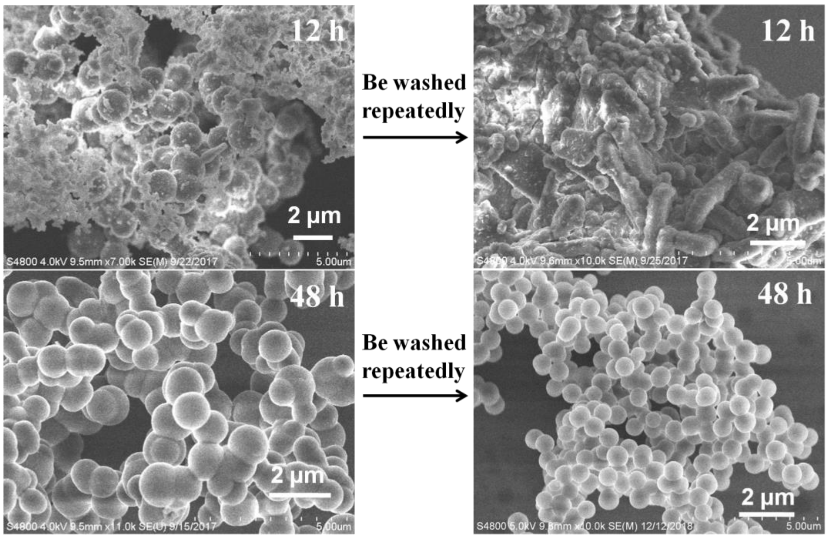

Supplementary Figure 22. FE-SEM images of 3D-Sp-COF@12 h and 3D-Sp-COF@48 h before and after being washed repeatedly.

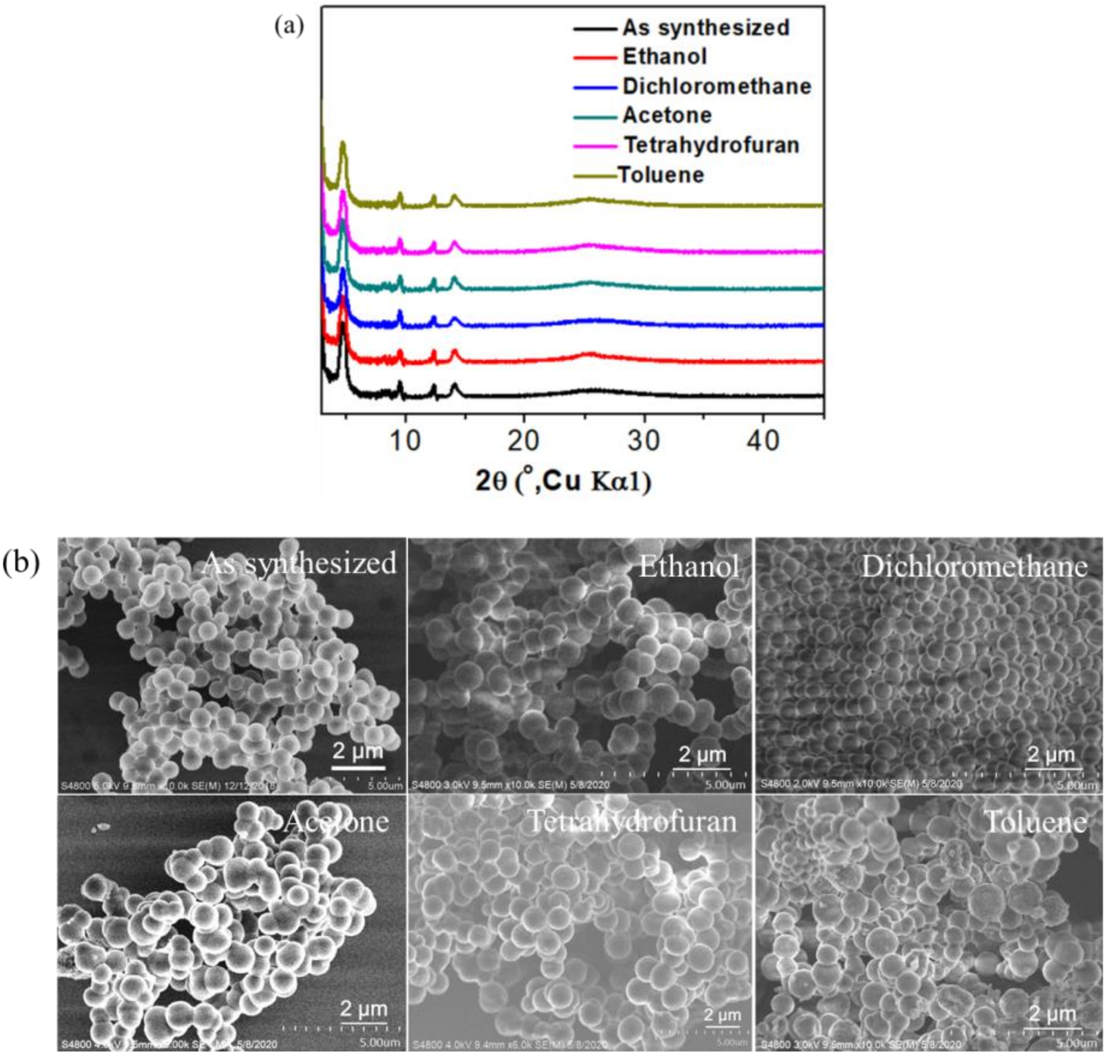

Supplementary Figure 23. PXRD profiles (a) and SEM images (b) of 3D-Sp-COF after treatment with ethanol, dichloromethane, acetone, tetrahydrofuran, and toluene, respectively.

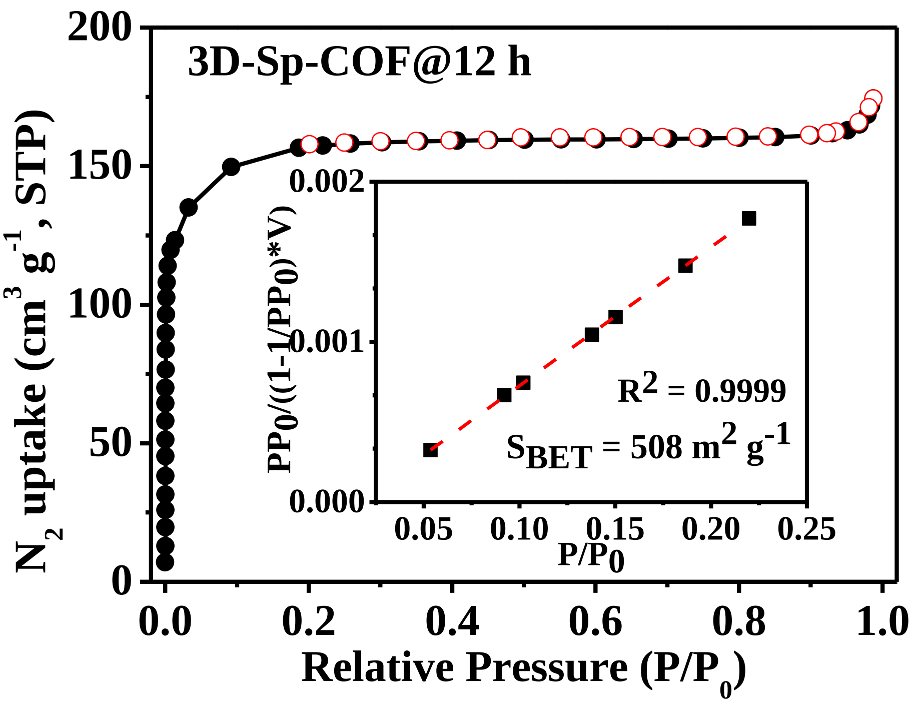

Supplementary Figure 24. N_2 adsorption-desorption isotherms of 3D-Sp-COF@12 h. Inset: BET surface area plots calculated from adsorption data.

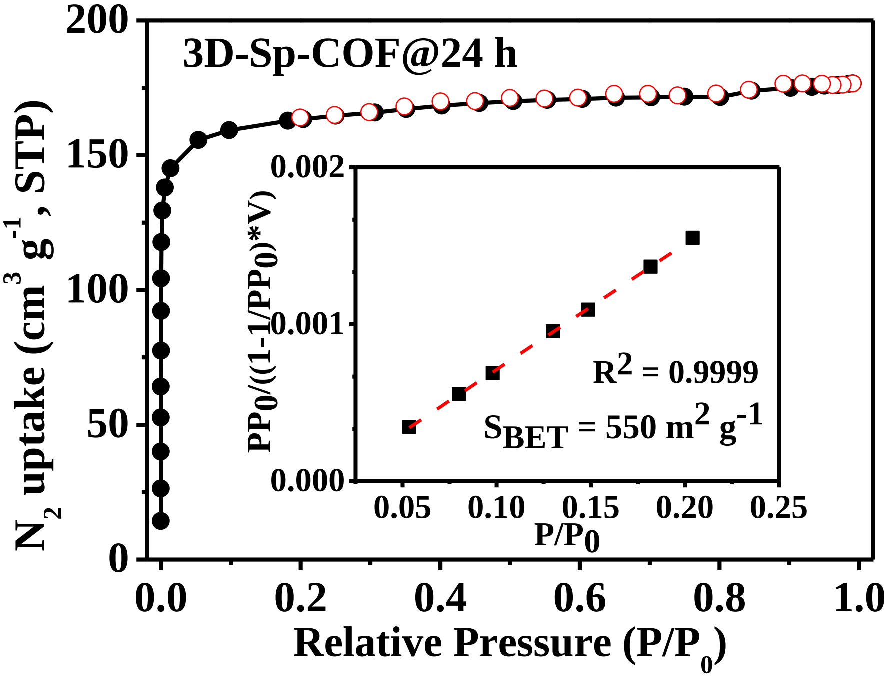

Supplementary Figure 25. N_2 adsorption-desorption isotherms of 3D-Sp-COF@24 h. Inset: BET surface area plots calculated from adsorption data.

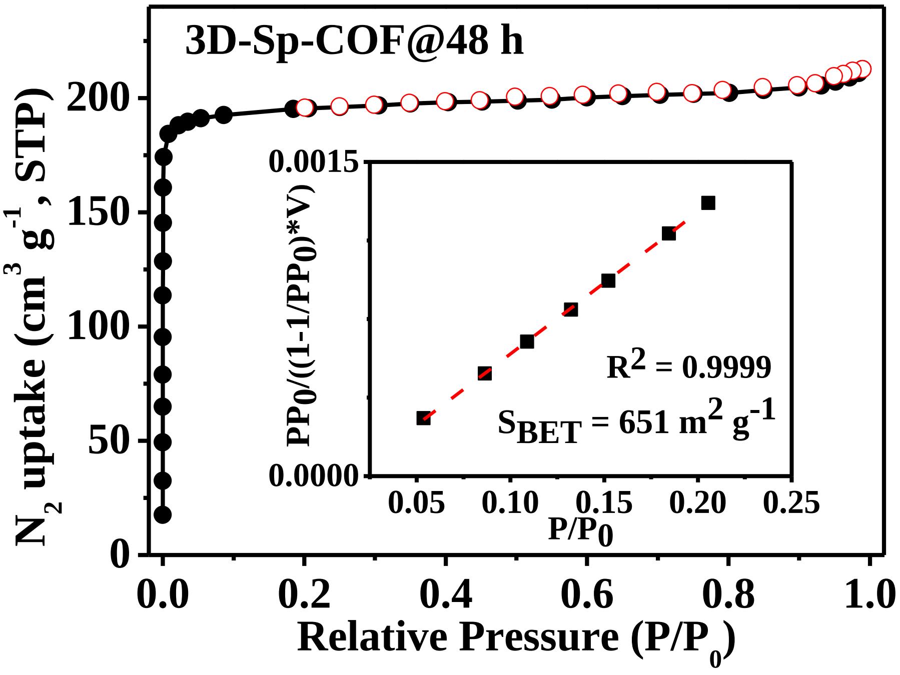

Supplementary Figure 26. N_2 adsorption-desorption isotherms of 3D-Sp-COF@48 h. Inset: BET surface area plots calculated from adsorption data.

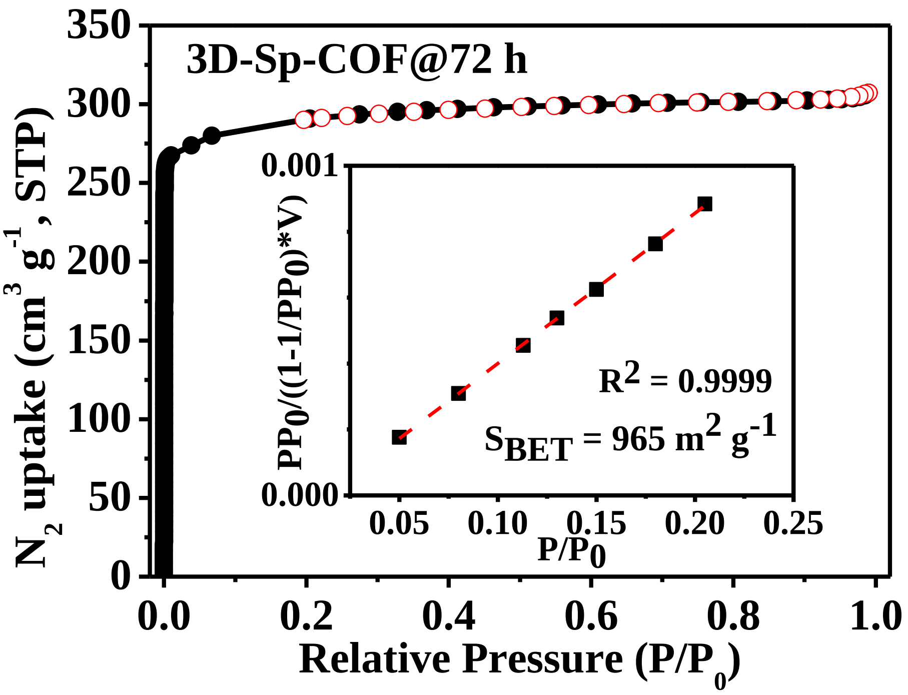

Supplementary Figure 27. N_2 adsorption-desorption isotherms of 3D-Sp-COF@72 h. Inset: BET surface area plots calculated from adsorption data.

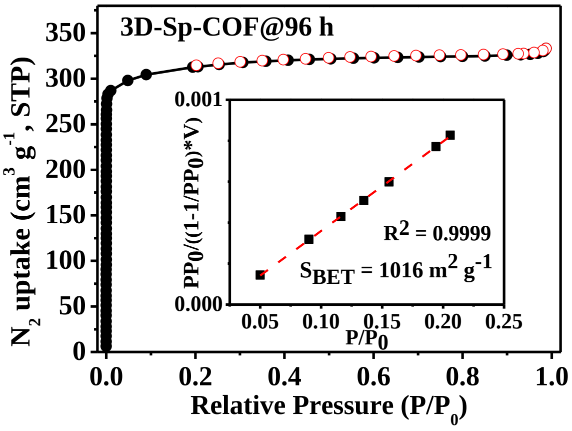

Supplementary Figure 28. N_2 adsorption-desorption isotherms of 3D-Sp-COF@96 h. Inset: BET surface area plots calculated from adsorption data.

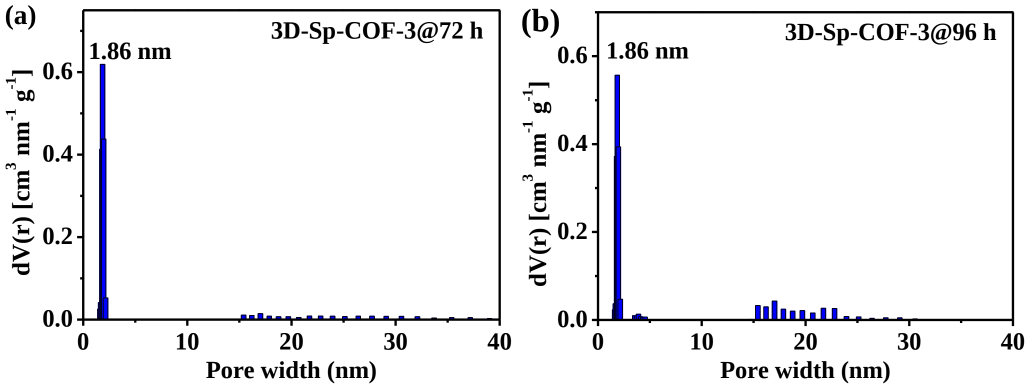

Supplementary Figure 29. Pore size distribution (PSD) of **(a)** 3D-Sp-COF@72 h and **(b)** 3D-Sp-COF@96 h.

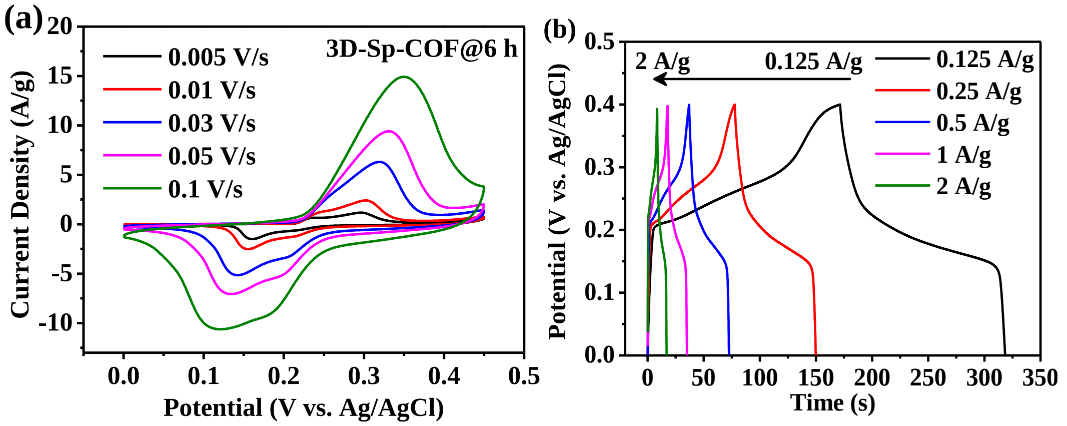

Supplementary Figure 30. Electrochemical performance of 3D-Sp-COF@6 h**. (a)** CVs at different scan rates (5, 10, 30, 50 and 100 mV s^{-1}); **(b)** GCDs at different current densities (0.125, 0.25, 0.5, 1 and 2 A g^{-1}).

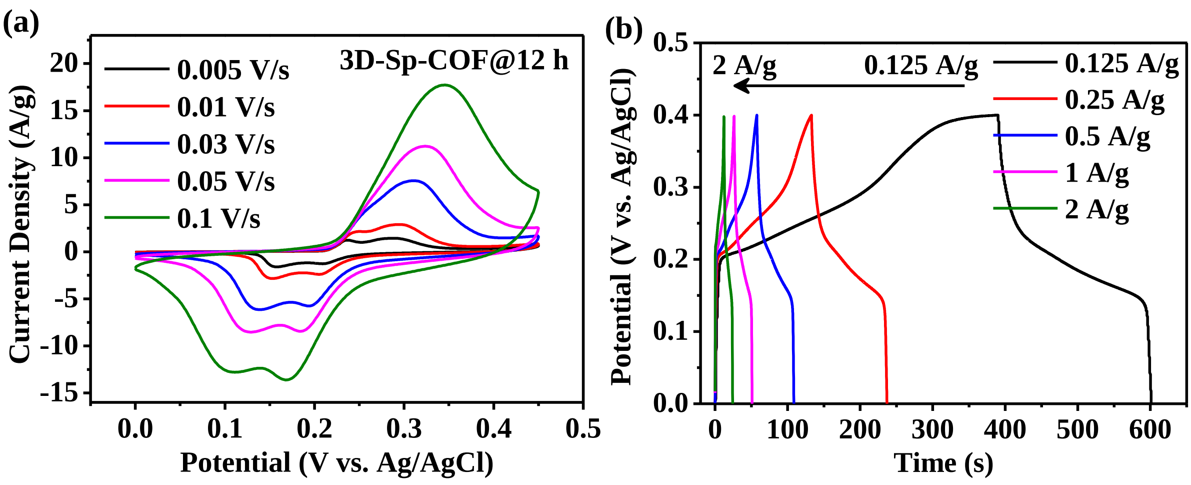

Supplementary Figure 31. Electrochemical performance of 3D-Sp-COF@12 h**. (a)** CVs at different scan rates (5, 10, 30, 50 and 100 mV s^{-1}); **(b)** GCDs at different current densities (0.125, 0.25, 0.5, 1 and 2 A g^{-1}).

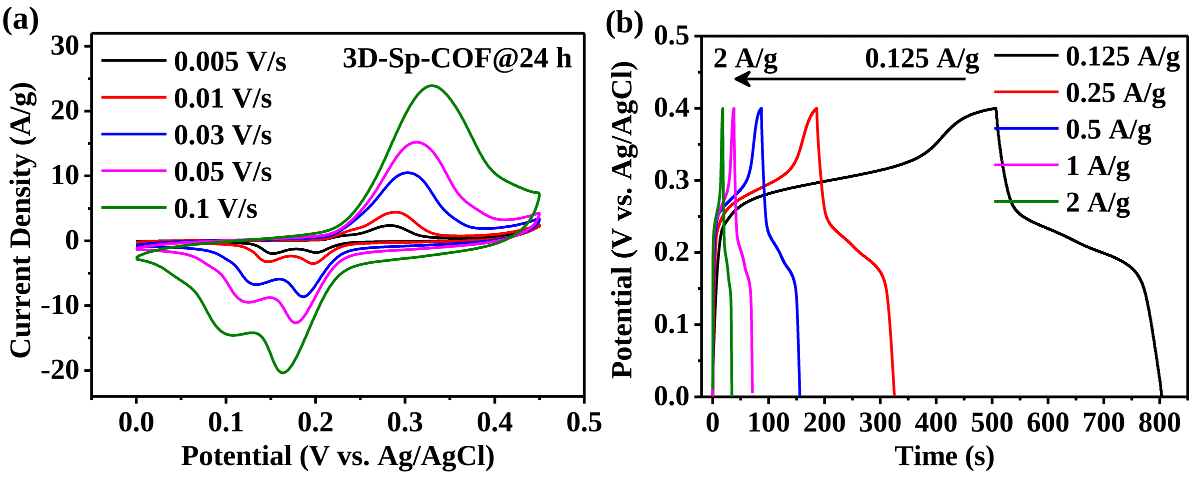

Supplementary Figure 32. Electrochemical performance of 3D-Sp-COF@24 h. **(a)** CVs at different scan rates (5, 10, 30, 50 and 100 mV s^{-1}); **(b)** GCDs at different current densities (0.125, 0.25, 0.5, 1 and 2 A g^{-1}).

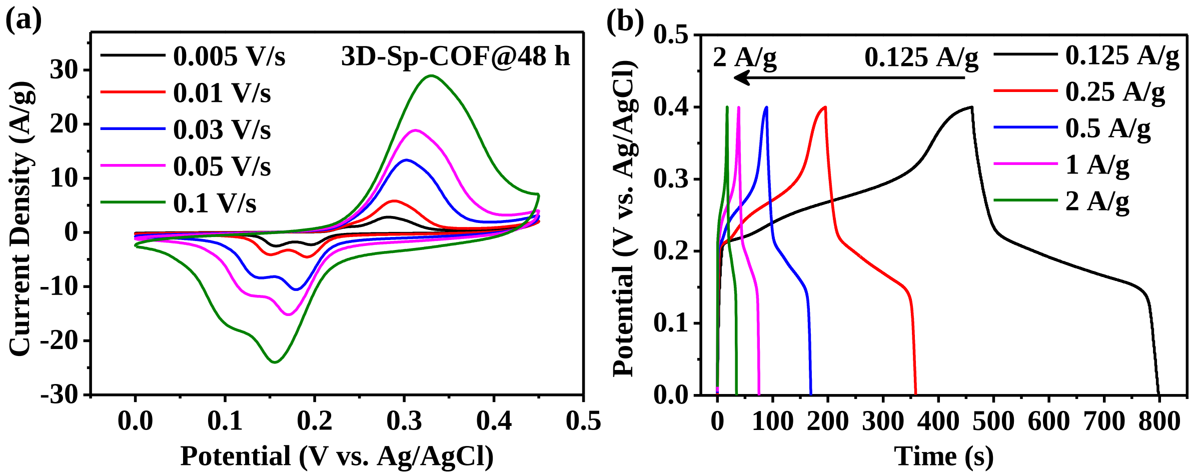

Supplementary Figure 33. Electrochemical performance of 3D-Sp-COF@48 h. **(a)** CVs at different scan rates (5, 10, 30, 50 and 100 mV s^{-1}); **(b)** GCDs at different current densities (0.125, 0.25, 0.5, 1 and 2 A g^{-1}).

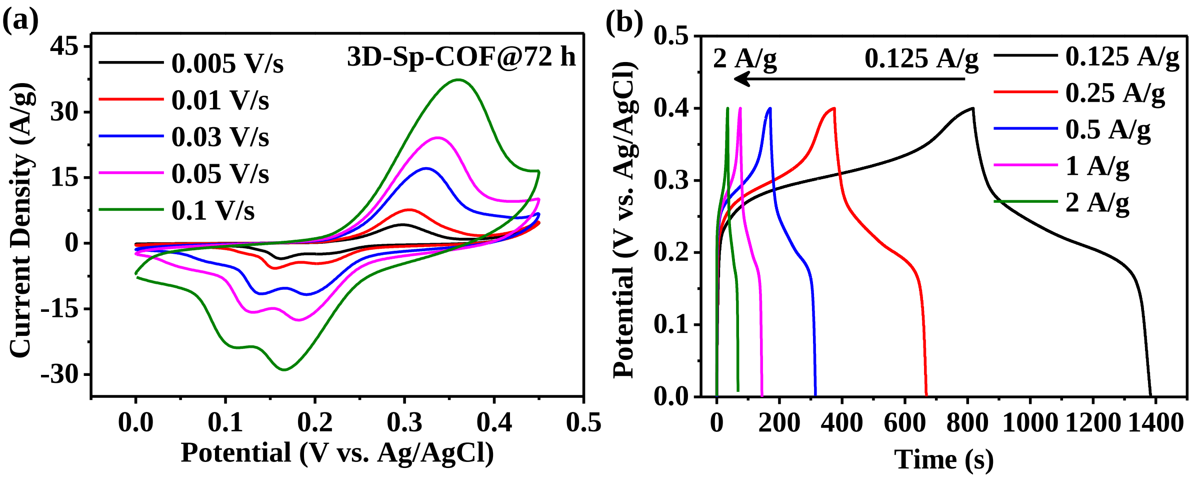

Supplementary Figure 34. Electrochemical performance of 3D-Sp-COF@72 h**. (a)** CVs at different scan rates (5, 10, 30, 50 and 100 mV s^{-1}); **(b)** GCDs at different current densities (0.125, 0.25, 0.5, 1 and 2 A g^{-1}).

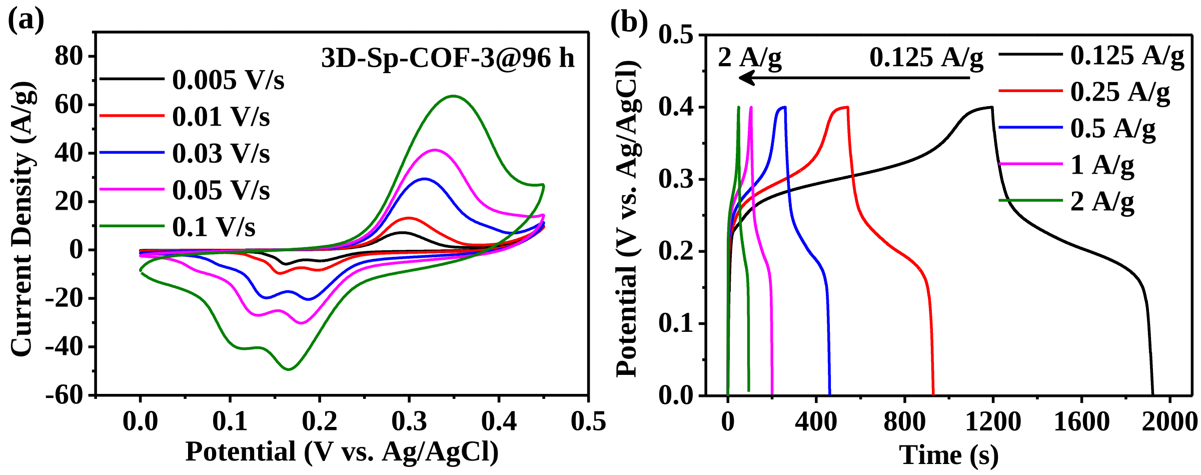

Supplementary Figure 35. Electrochemical performance of 3D-Sp-COF@96 h**. (a)** CVs at different scan rates (5, 10, 30, 50 and 100 mV s^{-1}); **(b)** GCDs at different current densities (0.125, 0.25, 0.5, 1 and 2 A g^{-1}).

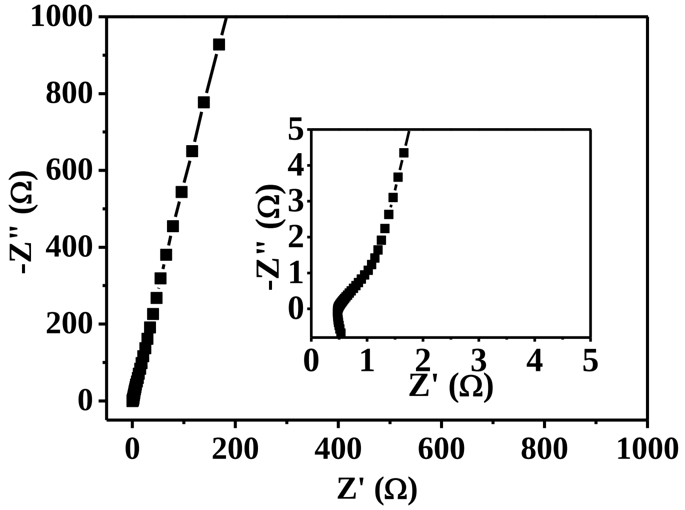

Supplementary Figure 36. Nyquist plots of EIS results collected at 0 V vs. Ag/AgCl for 3D-Sp-COF@96 h. Inset: close-up view on high frequency regions.

3. Supplementary Tables

Supplementary Table 1. The related parameters and t^+ of the composite electrolyte membranes.

Samples	R_{Io} (Ω)	R_{Is} (Ω)	I_o (μA)	I_s (μA)	t_{Li}^+
3D-Sp-COF	88	86	82.7	74.7	0.7

Supplementary Table 2. Theoretical elucidation of Li-ion migration behaviors based on **3D-Sp-COF** with different transmission pathways.

	X-axis pathway	Y-axis pathway	Z-axis pathway
Initial state (IS, kcal mol^{-1})	-6818643.94	-6818608.67	-6818643.60
Final states (FS, kcal mol^{-1})	-6818622.44	-6818581.50	-6818651.01
Transition state (TS, kcal mol^{-1})	-6818608.80	-6818556.56	-6818615.71
Location of transition state	0.58755	0.60038	0.40577
Energy of reaction (kcal mol^{-1})	21.50	27.17	-7.40
Energy of barrier (kcal mol^{-1})	35.14	52.11	27.90
Diffusion coefficient (D, cm^2 s^{-1})	6.52*10^{-28}	2.19*10^{-40}	6.78*10^{-23}
Lithium ion mobility (μ, cm^2 s^{-1} V^{-1})	2.54*10^{-26}	8.54*10^{-39}	2.64*10^{-21}

Supplementary Table 3. Comparison of gravimetric capacitances (F g^{-1}) with state-of-art promising electrode materials made up of COFs.

Electrode material	Gravimetric capacitance (F g^{-1})	Electrolyte	Scan rate/ current density	Cell Configuration	References
3D-Sp-COF@96 h	251	6 M KOH	0.5 A g^{-1}	three electrode	This work
TpOMe-DAQ	169	3 M H_2SO_4	0.35 A g^{-1}	three electrode	*J. Am. Chem. Soc.* **2018**, *140*, 10941
DqTp COF	154	1 M H_2SO_4	1.56 mA cm^{-2}	three electrode	*ACS Appl. Mater. Interfaces* **2018**, *10*, 28139
v-CNS-RGO-3	170	6 M KOH	1 A g^{-1}	two electrode	*Angew. Chem. Int. Ed.* **2018**, *57*, 1034
TpPa-$(OH)_2$	416	1 M phosphate buffer (pH 7.2)	0.5 A g^{-1}	three electrode	*Chem. Mater.* **2017**, *29*, 2074
COF-1-Fe^{III}<br>COF-1-Co^{II}<br>COF-1-Ni^{II}	460<br>160<br>125	6 M KOH	1 A g^{-1}	three electrode	*J. Mater. Chem. A* **2017**, *5*, 4343
TaPa-Py COF<br>DAB-TFP COF	209<br>98	1 M H_2SO_4	0.5 A g^{-1}	three electrode	*J. Mater. Chem. A* **2016**, *4*, 16312
BC-MS-700-14	160	1 M H_2SO_4	10 mV s^{-1}	two electrode	*J. Mater. Chem. A* **2016**, *4*, 4273
[TEMPO]100%-NiP-COF	167	0.1 M $(C_4H_9)_4NClO_4$ in CH_3CN	0.1 A g^{-1}	three electrode	*Angew. Chem. Int. Ed.* **2015**, *54*, 6814
DAAQ-TFP	48±10	1 M H_2SO_4	10 mV s^{-1}	three electrode	*J. Am. Chem. Soc.* **2013**, *135*, 16821

Supplementary Table 4. Comparison of gravimetric capacitance (F g^{-1}) with state-of-art promising electrode materials.

Active material	Gravimetric capacitance (F g^{-1})	Electrolyte	Scan rate/ current density	References
3D-Sp-COF@96 h	251	6 M KOH	0.5 A g^{-1}	This work
$Ni_3(HITP)_2$ MOF	111	$TEABF_4$/ACN	0.05 A g^{-1}	*Nat. Mater.* **2017**, *16*, 220
Triazatruxene conjugated microporous polymer (TAT-CMP-1)	141	1 M Na_2SO_4	1 A g^{-1}	*Chem. Sci.* **2017**, *8*, 2959
Porous triazine-based frameworks (PTF@700°C)	151	1-ethyl-3-methylimidazoliu m tetrafluoroborate	0.1 A g^{-1}	*J. Am. Chem. Soc.* **2015**, *137*, 219
ZIF-derived porous carbon	228	6 M KOH	0.1 A g^{-1}	*J. Mater. Chem. A* **2014**, *2*, 12873
Hierarchically porous B-doped carbons	160	1 M H_2SO_4	10 mV s^{-1}	*J. Mater. Chem. A* **2016**, *4*, 4273
GNrib	168	1 M H_2SO_4	1 A g^{-1}	*Nat. Chem.* **2016**, *8*, 718
NPC-F	284	6 M KOH	1 A g^{-1}	*Adv. Mater.* **2016**, *28*, 1981
CM-NF	189	6 M KOH	0.1 A g^{-1}	*Nat. Commun.* **2015**, *6*, 8503
PTF-700	151.3	$EMIMBF_4$	0.1 A g^{-1}	*J. Am. Chem. Soc.* **2015**, *137*, 219
N-doped carbon nanotube	215	1 M H_2SO_4	0.2 A g^{-1}	*ACS Appl. Mater. Interfaces* **2015**, *7*, 20083
Carbon-L-950	228	6 M KOH	0.1 A g^{-1}	*Adv. Mater.* **2014**, *26*, 2047-2051.
Porous N-doped hollow carbon spheres	213	6 M KOH	0.5 A g^{-1}	*J. Mater. Chem. A* **2014**, *2*, 5352
Three-dimensional (3D) honeycomb-like porous carbon	342	6 M KOH	0.2 A g^{-1}	*Nanoscale* **2014**, *6*, 13831
2D quasi-ordered nitrogen-enriched porous carbon nanohybrids	426	6 M KOH	1 A g^{-1}	*Nanoscale* **2016**, *8*, 10166
Graphene (activated)	200	$BMIM_4$/AN	0.7 A g^{-1}	*Science* **2011**, *332*, 1537

Supplementary Table 5. Unit cell parameters and fractional atomic coordinates for 3D-Sp-COF based on the non-interpenetrated dia topology.

Space group	I41/AMD		
Unit cell parameters	a = b = 39.492 Å, c = 83.904 Å and $\alpha = \beta = \gamma = 90.0°$		
**Atom**	**x**	**y**	**z**
C	-0.50000	0.22047	0.63564
C	-0.50000	0.28267	0.63122
C	-0.50000	0.25397	0.64160
H	-0.50000	0.30772	0.63647
H	-0.50000	0.25811	0.65435
H	-0.50000	0.07291	0.67749
C	-0.50000	0.18853	0.64628
N	-0.50000	0.18848	0.66204
C	-0.50000	0.15987	0.67350
C	-0.50000	0.16717	0.68990
C	-0.50000	0.14158	0.70176
C	-0.50000	0.10630	0.69815
C	-0.50000	0.09892	0.68146
C	-0.50000	0.12499	0.66944
H	-0.50000	0.19340	0.69366
H	-0.50000	0.11771	0.65701
C	-0.50000	0.07857	0.71168
C	-0.50000	0.04252	0.70882
C	-0.50000	0.01884	0.72186
C	-0.50000	0.02969	0.73780
C	-0.50000	0.06442	0.74089
C	-0.50000	0.08813	0.72818
H	-0.50000	0.15060	0.71385
H	-0.50000	0.03278	0.69680
H	-0.50000	0.11426	0.73177
H	-0.50000	0.07329	0.75309
C	-0.50000	0.00000	0.75000

Supplementary Table 6. Unit cell parameters and fractional atomic coordinates for 3D-Sp-COF based on the 2-fold-interpenetrated dia topology.

Space group	P42/N		
Unit cell parameters	a = b = 27.940 Å, c = 41.934 Å and $\alpha = \beta = \gamma = 90.0°$		
**Atom**	**x**	**y**	**z**
C	-0.22964	0.22572	1.22200
C	-0.20492	0.22802	1.25139
C	-0.22482	0.25166	1.27859
H	-0.17001	0.21151	1.25346
H	-0.20421	0.25225	1.30045
C	-0.07684	0.07764	1.07834
C	-0.08790	0.08859	1.04584
C	-0.06497	0.06538	1.01949
C	-0.02987	0.03008	1.02464
C	-0.01853	0.01873	1.05631
C	-0.04108	0.04152	1.08288
H	-0.11402	0.11599	1.04051
H	-0.03114	0.03060	1.10670
C	-0.10180	0.10386	1.10684
C	-0.07992	0.10768	1.13728
C	-0.10215	0.13180	1.16333
C	-0.14762	0.15372	1.16085
C	-0.17004	0.15026	1.13063
C	-0.14769	0.12602	1.10438
H	-0.04475	0.09264	1.14115
H	-0.08331	0.13350	1.18587
H	-0.20496	0.16618	1.12716
H	-0.16713	0.12425	1.08212
C	-0.21011	0.20077	1.19174
N	-0.16855	0.17832	1.18901
H	-0.07454	0.07517	0.99535
C	0.00000	0.00000	1.00000

Supplementary Table 7. Unit cell parameters and fractional atomic coordinates for 3D-Sp-COF based on the 3-fold-interpenetrated dia topology.

Space group	I41/AMD		
Unit cell parameters	a = b = 39.492 Å, c = 27.968 Å and $\alpha = \beta = \gamma = 90.0°$		
**Atom**	**x**	**y**	**z**
C	0.00000	0.22408	1.16217
C	0.00000	0.21593	1.11268
C	0.00000	0.25883	1.17366
H	0.00000	0.18961	1.10184
H	0.00000	0.26661	1.21077
H	0.00000	0.08767	1.29658
C	0.00000	0.19559	1.19946
N	0.00000	0.19968	1.24650
C	0.00000	0.17298	1.28379
C	0.00000	0.18145	1.33310
C	0.00000	0.15627	1.36999
C	0.00000	0.12110	1.35951
C	0.00000	0.11325	1.30957
C	0.00000	0.13815	1.27321
H	0.00000	0.17026	1.18494
H	0.00000	0.20789	1.34329
H	0.00000	0.12942	1.23664
C	0.00000	0.09263	1.39855
C	0.00000	0.05865	1.38429
C	0.00000	0.03228	1.41702
C	0.00000	0.03434	1.46702
C	0.00000	0.06873	1.48296
C	0.00000	0.09715	1.44967
H	0.00000	0.16467	1.40677
H	0.00000	0.05070	1.34763
H	0.00000	0.12247	1.46434
H	0.00000	0.07380	1.52098
C	0.00000	0.00000	1.50000

Supplementary Table 8. Unit cell parameters and fractional atomic coordinates for 3D-Sp-COF based on the 4-fold-interpenetrated dia topology.

Space group	P4/N		
Unit cell parameters	a = b = 27.822 Å, c = 21.030 Å and $\alpha = \beta = \gamma = 90.0°$		
**Atom**	x	y	z
C	-0.76115	0.73266	2.43878
C	-0.77375	0.77971	2.45646
H	-0.93240	0.71726	2.21137
C	-0.77490	0.71264	2.37645
N	-0.80127	0.73710	2.33697
C	-0.82205	0.71752	2.28010
C	-0.80166	0.67800	2.24813
C	-0.82922	0.65153	2.20501
C	-0.87715	0.66435	2.19347
C	-0.89522	0.70725	2.21909
C	-0.86747	0.73402	2.26162
H	-0.76699	0.67513	2.36736
H	-0.76587	0.66552	2.25967
H	-0.88337	0.76471	2.28529
C	-0.91111	0.62643	2.17514
C	-0.93572	0.60323	2.22419
C	-0.96401	0.56358	2.21130
C	-0.97007	0.54618	2.14845
C	-0.94627	0.57084	2.09886
C	-0.91633	0.61028	2.11228
H	-0.81489	0.61826	2.18608
H	-0.93169	0.61479	2.27308
H	-0.89694	0.62773	2.07408
H	-0.94982	0.55843	2.05030
C	-0.70318	0.26269	2.51719
H	-0.66668	0.27247	2.52965
H	-0.80318	0.20760	2.57637
C	-1.00000	0.50000	2.13407

Supplementary Table 9. Unit cell parameters and fractional atomic coordinates for 3D-Sp-COF based on the 5-fold-interpenetrated dia topology.

Space group	I41/AMD		
Unit cell parameters	a = b = 39.484 Å, c = 16.773 Å and $\alpha = \beta = \gamma = 90.0°$		
**Atom**	**x**	**y**	**z**
C	0.00000	0.72813	-0.30431
C	0.00000	0.57730	0.05403
C	0.00000	0.58820	0.13561
C	0.00000	0.56513	0.20135
C	0.00000	0.52996	0.18857
C	0.00000	0.51863	0.10958
C	0.00000	0.54123	0.04316
H	0.00000	0.61478	0.15025
H	0.00000	0.53030	-0.01567
C	0.00000	0.60296	-0.01777
C	0.00000	0.59206	-0.09865
C	0.00000	0.61507	-0.16400
C	0.00000	0.65054	-0.15369
C	0.00000	0.66206	-0.07384
C	0.00000	0.63895	-0.00791
H	0.00000	0.56548	-0.11331
H	0.00000	0.60489	-0.22393
H	0.00000	0.68901	-0.06194
H	0.00000	0.64996	0.05093
C	0.00000	0.70643	-0.22781
N	0.00000	0.67296	-0.22462
C	0.00000	0.71462	0.61766
C	0.00000	0.73598	0.54890
H	0.00000	0.72422	0.49058
H	0.00000	0.68742	0.60950
C	0.00000	0.50000	0.25000

Supplementary Table 10. Unit cell parameters and fractional atomic coordinates for 3D-Sp-COF based on the 6-fold-interpenetrated dia topology.

Space group	P42/NNM		
Unit cell parameters	a = b = 27.269 Å, c = 14.200 Å and $\alpha = \beta = \gamma = 90.0°$		
**Atom**	**x**	**y**	**z**
C	0.21484	0.21484	0.72879
C	0.23978	0.23978	0.65502
H	0.18764	0.18764	0.70986
H	0.23095	0.23095	0.58266
C	0.22441	0.22441	-0.17461
H	0.08789	0.08789	0.09835
C	0.19618	0.19618	-0.09882
N	0.20147	0.20147	-0.00664
C	0.17487	0.17487	0.06863
C	0.18437	0.18437	0.16521
C	0.15917	0.15917	0.23936
C	0.12301	0.12301	0.22066
C	0.11422	0.11422	0.12282
C	0.13907	0.13907	0.04989
H	0.16996	0.16996	-0.12534
H	0.21161	0.21161	0.18363
H	0.12947	0.12947	-0.02148
C	0.09437	0.09437	0.29880
C	0.05952	0.05952	0.27177
C	0.03289	0.03289	0.33686
C	0.03519	0.03519	0.43508
C	0.07051	0.07051	0.46576
C	0.09942	0.09942	0.39938
H	0.16829	0.16829	0.31135
H	0.05091	0.05091	0.20001
H	0.12547	0.12547	0.42748
H	0.07595	0.07595	0.54052
C	0.00000	0.00000	0.50000

Supplementary Table 11. Unit cell parameters and fractional atomic coordinates for 3D-Sp-COF based on the 7-fold-interpenetrated dia topology.

Space group	I41/AMD		
Unit cell parameters	a = b = 35.554 Å, c = 11.351 Å and $\alpha = \beta = \gamma = 90.0°$		
**Atom**	**x**	**y**	**z**
C	0.50000	0.73197	1.23150
C	0.50000	0.58224	1.74505
C	0.50000	0.59323	1.86337
C	0.50000	0.56788	1.95544
C	0.50000	0.53177	1.91871
C	0.50000	0.48017	1.80820
C	0.50000	0.54444	1.72123
H	0.50000	0.62142	1.88532
H	0.50000	0.53305	1.63820
C	0.50000	0.60892	1.64545
C	0.50000	0.59637	1.53200
C	0.50000	0.61974	1.43984
C	0.50000	0.65690	1.45396
C	0.50000	0.67061	1.56495
C	0.50000	0.64696	1.65924
H	0.50000	0.56842	1.50922
H	0.50000	0.60854	1.35541
H	0.50000	0.69941	1.57898
H	0.50000	0.65890	1.74222
C	0.50000	0.71371	1.34428
N	0.50000	0.67876	1.35276
C	0.50000	0.50000	2.00000
H	0.50000	0.57620	2.04310
C	0.50000	0.78761	1.12048
C	0.50000	0.76977	1.22539
H	0.50000	0.81676	1.11938
H	0.50000	0.78560	1.30214

Supplementary Table 12. Unit cell parameters and fractional atomic coordinates for 3D-SpOH-COF based on the 7-fold-interpenetrated dia topology.

Space group	I41/AMD		
Unit cell parameters	a = b = 41.6510 Å, c = 9.9993 Å and $\alpha = \beta = \gamma = 90.0°$		
**Atom**	**x**	**y**	**z**
C	0.50000	0.71779	1.18017
C	0.50000	0.57275	1.69245
C	0.50000	0.58238	1.82984
C	0.50000	0.55997	1.93641
C	0.50000	0.52699	1.90999
C	0.50000	0.48360	1.78154
C	0.50000	0.53867	1.66605
H	0.50000	0.60731	1.85847
H	0.50000	0.52787	1.56808
C	0.50000	0.59522	1.58221
C	0.50000	0.5843	1.44742
C	0.50000	0.60534	1.34001
C	0.50000	0.6383	1.35867
C	0.50000	0.65033	1.48975
C	0.50000	0.62914	1.60000
H	0.50000	0.55958	1.41797
H	0.50000	0.59571	1.23962
H	0.50000	0.67574	1.50815
H	0.50000	0.64005	1.69769
C	0.50000	0.68779	1.22108
N	0.50000	0.65749	1.23927
H	0.5000	0.56834	2.03859
C	0.50000	0.77685	1.21615
C	0.50000	0.74606	1.27092
H	0.50000	0.79735	1.28316
O	0.50000	0.74331	1.39789
